# Persistent High-Risk HPV Positivity Without Confirmed CIN2+: Diagnostic Uncertainty, Risk Stratification, and Management—A Narrative Review

**DOI:** 10.3390/diagnostics16213482

**Published:** 2026-10-24

**Authors:** Sandra Teodora Gavril, Daniela Roxana Matasariu, Ioana Sadiye Scripcariu, Iustina Petra Solomon Condriuc, Razvan Popovici, Demetra Socolov, Alexandru Carauleanu

**Affiliations:** 1 “Grigore T. Popa” University of Medicine and Pharmacy, 16 University Street, 700115 Iasi, Romaniademetra.socolov@umfiasi.ro (D.S.); ale.carauleanu@umfiasi.ro (A.C.); 2 Department of Obstetrics and Gynecology, Cuza Voda Hospital, 700038 Iasi, Romania

**Keywords:** human papillomavirus, HPV persistence, cervical cancer screening, risk stratification, colposcopy, diagnostic accuracy, molecular triage, biomarkers, extended genotyping, risk-based management

## Abstract

Primary high-risk human papillomavirus (hrHPV) testing detects cervical precancer earlier than cytology and, by design, generates a large population of virus-positive women in whom high-grade disease is not confirmed. Where the infection persists, the clinician must judge whether a negative or non-diagnostic assessment reflects absence of disease or failure of detection. This review asks what that state represents, which tools reclassify absolute CIN3+ risk, and how surveillance and intervention should be balanced. This narrative review synthesises relevant evidence from published studies and international guidance on persistent hrHPV infection, diagnostic assessment, risk stratification and management, with reporting guided by the Scale for the Assessment of Narrative Review Articles (SANRA). Persistent hrHPV positivity without confirmed CIN2+ is a heterogeneous risk state, not a diagnosis, and covers three clinical situations that should not be conflated. Absolute CIN3+ risk is set principally by genotype and by the duration of type-specific persistence; genotype alone spans an approximately twenty-fold range of immediate risk in hrHPV-positive screening populations. Pooled colposcopic sensitivity for CIN2+ was approximately 75% in heterogeneous referral populations, while one selected retrospective series of cytology-negative hrHPV-positive women reported sensitivity near 50%. Selected referral cohorts have reported CIN2+/HSIL detection yields of approximately 8% to 14% among persistently hrHPV-positive women with normal cytology; these composite, selection-dependent estimates should not be read as the prevalence of occult CIN3+. Extended genotyping and p16/Ki-67 dual-stain cytology carry guideline-adopted management rules, although validation specifically after documented persistence remains limited. Controlled evidence for treating persistent hrHPV without confirmed CIN2+ is sparse and conflicting. Recent randomised trials of vaginal interventions report discordant short-term virological results, and none establishes durable clearance or a reduction in CIN3+ risk. Short-term loss of HPV detectability is not equivalent to prevention of precancer, and management should remain risk-based.

## 1. Introduction

Cervical cancer prevention has been reorganised around detection of the causal agent, not of its morphological consequences. In pooled follow-up of four European randomised screening trials, hrHPV-based screening was associated with a lower incidence of invasive cervical cancer than cytology (rate ratio 0.60, 95% CI 0.40–0.89; median follow-up 6.5 years) [1]. Most organised programmes in high-income settings have adopted it. The trade-off is specificity. Roughly 12% of women worldwide with normal cytology carry a cervical HPV infection of any type, the commonest genotypes being HPV16 (3.2%), HPV18 (1.4%) and HPV52 (0.9%) [2]. Most of these infections are productive and self-limiting. In the control arm of a large vaccine trial, 53% had cleared by 12 months and 79% by 24 months [3]. Programmes built on viral detection therefore generate, by design, a large population of women who are test-positive without confirmed high-grade cervical disease.

The clinical problem begins when the infection does not clear. Type-specific persistence is the necessary intermediate step between infection and precancer, and its prognostic weight is substantial. In a Danish population-based cohort followed for up to 13.4 years, the 12-year absolute risk of cervical intraepithelial neoplasia grade 3 or worse (CIN3+) was 26.7% (95% CI 21.1–31.8) after a single baseline HPV16 detection. It reached 47.4% (95% CI 34.9–57.5) when HPV16 was detected at two consecutive examinations [4]. Persistence and prevalent disease are nevertheless not the same thing. When women referred for persistent hrHPV with normal cytology undergo colposcopy, high-grade disease is histologically confirmed in a minority. High-grade squamous intraepithelial lesion (HSIL) was found on histology in 46 of 337 women (13.6%) in a retrospective Finnish screening-programme cohort [5]. CIN2 or worse (CIN2+) was found in 7.6% of cytology-negative women in a German series, all of whom had a colposcopically directed biopsy or, for a type 3 transformation zone, endocervical curettage [6]. Among women aged 65 or older, it was found in 20 of the 182 with a biopsy or excision specimen [7]. Most such women therefore leave the colposcopy clinic with a positive viral test, no high-grade histological diagnosis, and no treatable target.

What should be done for them is unsettled, and the disagreement among high-income programmes is not marginal. Contemporary guidance ranges from repeat HPV-based testing at one year without colposcopy [8], through referral at the third consecutive positive test irrespective of cytology [9], to direct referral to colposcopy of HPV16- or HPV18-positive women regardless of cytology [10]. A separate, resource-stratified position is taken by the World Health Organization, which permits ablative treatment on the basis of a positive HPV test alone where triage cannot be guaranteed [11]. These positions rest on largely the same evidence base. They differ mainly in how they weigh occult disease against the harms of pursuing it and in the resources available to pursue it.

This review examines the evidence underlying that disagreement. The analysis is organised around three questions. First, what does persistent hrHPV positivity without confirmed CIN2+ actually represent, given that a negative or non-diagnostic assessment may reflect absence of disease, failure of detection, or an infection that is no longer biologically active? Second, which stratification tools genuinely reclassify a woman’s absolute risk of CIN3+, and which merely relabel it? Third, given controlled intervention evidence that is sparse and conflicting, what is the evidence-based balance between surveillance and intervention? Age, transformation-zone type, immune status and screening history each shift that balance. Primary screening interval selection, management of histologically confirmed CIN2+ and post-treatment follow-up are considered only where they bear on these questions.

Existing syntheses have addressed adjacent questions. The closest is a systematic review and meta-analysis of alternatives to surveillance, which screened 2135 records and included 12 studies, four cohorts and eight randomised trials, covering oral agents, topical agents, vaccination and non-surgical device treatments; it concluded that some interventions may bring forward resolution of persistent hrHPV and regression of low-grade CIN compared with natural clearance [12]. Its eligible population was heterogeneous, encompassing women with persistent hrHPV and women with low-grade disease up to CIN1, with variable diagnostic histories and persistence definitions; its pooled signal is therefore not specific to any one of the three clinical states defined here. Biomarker-focused reviews, including a recent narrative review in this journal, have separately mapped the diagnostic and prognostic performance of p16/Ki-67, extended genotyping, methylation, E6/E7 mRNA and related technologies across HPV-positive screening populations [13]. What has received comparatively limited integrated discussion is the clinical management problem itself: the woman who remains hrHPV-positive after a negative, non-diagnostic or non-high-grade assessment. This review is therefore organised around that problem rather than around the biomarkers, which are treated as instruments for allocating diagnostic intensity within it. Three bodies of evidence are usually kept apart: the residual absolute risk conferred by persistence, the measured failure rate of the examination used to exclude high-grade disease, and the sparse and conflicting evidence on intervention directed at the infection. Reconciling them is the aim here. Programme-level outcome data, interval-cancer rates and cost-effectiveness analyses fall outside that aim and are not reviewed.

### Narrative Review Methodology

This is a narrative review. It was not registered, and it does not follow systematic-review methodology. It draws on literature identified through non-exhaustive searches of PubMed/MEDLINE, Europe PMC and Scopus, supplemented by international guidelines and consensus documents and by the reference lists of these documents and of recent reviews. Priority was given to evidence addressing HPV persistence, diagnostic assessment, risk stratification and interventions in women without confirmed CIN2+. Five tables are provided in the Supplementary Materials. Table S1 summarises the longitudinal risk evidence, Table S2 the detection yield of colposcopic and histological assessment and Table S3 the intervention evidence, while Tables S4 and S5 document the methods described here. Table S4 summarises the literature sources and principal search concepts. Reporting was guided by the Scale for the Assessment of Narrative Review Articles (SANRA), with an item-by-item mapping provided in Table S5.

Sources were selected for relevance to the three questions stated above, rather than through a systematic eligibility and screening process. Current guidelines and consensus statements, longitudinal cohorts with histological endpoints, randomised trials and diagnostic-accuracy studies with a defined reference standard were given priority; high-quality systematic reviews were used where they summarise a body of evidence more reliably than any single study. Seminal earlier work was retained where it remains the primary source of a natural-history estimate. Primary studies were cited for individual-study estimates, whereas systematic reviews and meta-analyses were cited for pooled estimates. No risk-of-bias instrument was applied and no quantitative pooling was undertaken. Statements about the absence of evidence therefore describe what these searches identified. They are not proof that no such study exists.

## 2. Defining the Clinical Entity

### 2.1. What Counts as Persistence

No agreed operational definition of hrHPV persistence exists, and the variation is large enough to alter the apparent magnitude of risk. A systematic review of 41 studies including more than 22,500 women found relative risks for cervical neoplasia associated with persistence ranging from 1.3 (95% CI 1.1–1.5) to 813.0 (95% CI 168.2–3229.2) [14]. The higher estimates came from studies that required documentation beyond 12 months, used longer testing intervals, or used HPV-negative referents in place of HPV-positive ones. Most defined persistence simply as positivity at two or more timepoints, without specifying genotype [14].

The choice between any-type and type-specific persistence and the choice of interval are not neutral. In a nested analysis within the ASCUS-LSIL Triage Study, repeat detection of any hrHPV at 12 months had a sensitivity of 90.1% and a specificity of 66.7% for incident CIN2/3. At 24 months, sensitivity fell to 77.0% while specificity rose to 80.1%. Requiring the same genotype at both timepoints reduced sensitivity by about 5% at 12 months and by more than 20% at 24 months, while improving specificity by roughly 10% [15]. Very short intervals perform worst of all. In the multicentre ESTAMPA study, 61% of 5390 hrHPV-positive women remained positive when retested at a median of 1.8 months. Sensitivity for CIN3+ was 81.5–87.7% depending on assay, but specificity remained below 50% and referral rates were 57.4% and 68.2% [16]. Retesting at a short interval largely re-detects the same prevalent infection; it does not identify a distinct biological state.

Throughout this review, persistence means type-specific detection of the same genotype at two visits separated by at least 12 months. That definition is adopted for comparability with the risk estimates on which management thresholds are based and because type-specific persistence is the biological process that precedes transformation. The sensitivity penalty documented above means it is not the most inclusive definition. Several of the cohorts cited below, and every screening programme compared in Section 6 that acts on repeated HPV positivity, instead use any-type persistence, that is, consecutive positive results without regard to genotype. Where that is the case it is stated, and estimates generated under different definitions should not be treated as interchangeable.

### 2.2. What Counts as Absence of Confirmed High-Grade Disease

The phrase used in this review, persistent hrHPV positivity without confirmed CIN2+, conceals three clinically distinct states, and none of them is equivalent to a demonstrated absence of cervical disease. In state 1, a woman is hrHPV-positive with negative cytology and has not undergone colposcopy; the cervix has not been examined, and the absence of a lesion has not been established at all. In state 2, colposcopy has been performed, no acetowhite abnormality was identified and no histological result is available. In state 3, histological sampling has been performed and shows negative or inflammatory histology or CIN1, that is, less than CIN2, irrespective of whether the colposcopic impression was normal or abnormal; CIN1 is itself a cervical lesion, and what has been excluded is high-grade disease and not disease of any grade. Within state 3, two descriptive subcategories are distinguished: state 3a, negative or inflammatory histology without identified CIN, and state 3b, histologically confirmed CIN1. The three states are hierarchical and mutually exclusive. Where histological sampling is available, the woman is classified as state 3 regardless of the colposcopic impression and as state 3b if any sample shows CIN1. The endpoint excluded is stated wherever a figure is quoted.

These states carry materially different residual risks, and conflating them is a recurrent source of confusion. Among more than 20,000 women in whom colposcopy did not identify CIN2+, the subsequent five-year risk of CIN2+ varied with the referral result. It was 10% after an hrHPV-positive result with atypical squamous cells of undetermined significance or low-grade cytology, 16% after atypical squamous cells cannot exclude HSIL, and 24% after HSIL or worse [17]. A negative colposcopy is therefore informative only in the context of the result that prompted it, and only if the examination met accepted quality standards. Those standards include adequate assessment of the transformation zone and multiple targeted biopsies wherever any abnormality is seen [18].

The two subcategories of state 3 share the absence of confirmed CIN2+, but they are neither biologically nor morphologically equivalent, and histology conveys different diagnostic information in each. In state 3a, no CIN has been identified in the sampled tissue, a finding that establishes neither the absence of cervical HPV infection nor the absence of unsampled high-grade disease (Section 4). The natural history of state 3a is that of persistent infection without a demonstrated lesion (Section 3), which should not be equated with the course of a confirmed CIN1 lesion. In state 3b, histology demonstrates CIN1, a low-grade HPV-associated lesion generally reflecting productive infection and characterised by a substantial potential for regression [19]. The subdivision is descriptive and does not imply that state 3b carries a higher subsequent CIN3+ risk than state 3a under otherwise identical circumstances. The risk estimates quoted above come from a cohort that combined CIN1 with negative biopsy results because their findings were similar [17]. In either subcategory, subsequent management should be guided by the referral cytology and HPV result, genotype, screening history, adequacy of colposcopy and the applicable risk-based guidance (Section 6), not by the subdivision alone.

Nomenclature follows the source literature, which is not uniform. Findings are reported using the three-tiered CIN system or the two-tiered squamous intraepithelial lesion system as each primary study used them, HSIL corresponding to CIN2 and CIN3 combined [19]. The endpoint is named wherever a figure is quoted.

### 2.3. Scale of the Problem

In a United States cohort of nearly one million women screened by co-testing, hrHPV positivity with negative cytology accounted for 3.6% of screening tests, and 32,374 such women formed the analysis population. The five-year CIN3+ risk in this group was 4.5% (95% CI 4.2–4.8) and the five-year cancer risk 0.34% (95% CI 0.26–0.45). At the one-year return visit 47% remained hrHPV-positive, a proportion that varied little between ages 30 and 64 [20]. Because co-testing selects a cytology-negative subgroup from an already screened population, these figures understate the size of the group without confirmed CIN2+ generated by primary HPV screening, in which all hrHPV-positive women enter the pathway. In a Finnish population-based screening cohort of 35,084 genotyped women, 884 of 2006 hrHPV-positive women with negative or borderline cytology (44.1%) met a type-specific persistence definition, and 197 of these (22.3%) progressed to precancer. Persistence was most frequent with HPV52 (52.2%), HPV58 (50.5%), HPV31 (50.4%) and HPV16 (48.9%) [21]. Persistence is thus common. It is not rare even for genotypes of modest oncogenic potential, and it identified a group in which roughly one woman in five was found to have precancer within four years.

## 3. Natural History and the Determinants of Absolute Risk

### 3.1. Clearance Kinetics and the Shape of Risk over Time

Analysis of the control arm of a large vaccine trial gives the clearest description of clearance dynamics. Among 16,785 cervical HPV infections in 4825 women followed for 48 months, cumulative clearance was 53% at 12 months, 79% at 24, 87% at 36 and 89% at 48 months. Median time to clearance was 6.26 months for transient infections and 18.85 months for those already persistent at six months, and clearance was slower for HPV16 (median 17.11 months) than for other genotypes [3]. Approximately 80% of type-specific infections in an hrHPV-positive screening population were no longer detectable within three years [22]. The two differ because the trial figure counts all infections and the cohort figure counts type-specific infections in women selected for positivity. The clinically relevant population is defined by what remains after the first year, not by the prevalence of infection at any single test.

Incident detections behave differently from prevalent ones. In a seven-year population-based cohort, 66 of 85 cases of CIN2 or worse detected during follow-up arose from infections present at enrolment, and newly detected infections carried very low absolute risks irrespective of age [23]. Risk accrues to infections that have already demonstrated the capacity to persist. This is the principal justification for basing management on serial test results and not on a single one. Failure to clear is thought to reflect inadequate cell-mediated immunity. Regression of anogenital HPV lesions is accompanied by a CD4-positive, T-helper-1-dominated response, and failure to mount that response is the prevailing explanation for persistence. No immunological measurement currently has an established role in routine individual risk assignment [24].

### 3.2. Genotype Is a Major Axis of Risk

Of the variables routinely available at the point of care, genotype separates absolute risk most sharply. In a 16-year Taiwanese community cohort of 10,123 women with initially normal cytology, the cumulative risk of invasive cervical cancer was 13.5% for HPV16, 10.3% for HPV58, 4.0% for other carcinogenic types and 0.26% for HPV-negative women. Persistent carcinogenic infection carried a hazard ratio of 75.4 (95% CI 31.8–178.9) versus persistent negativity [25]. Extended-genotyping data from 14,158 type-specific infections in 11,573 hrHPV-positive women support a four-tier hierarchy. HPV16 stands alone, at a seven-year CIN3+ risk of 22%; HPV18 and HPV45 form the second tier and HPV31, 33, 35, 52 and 58 the third, both above 5%; HPV39, 51, 56, 59 and 68 fall below 5% [22]. Progression hazards for CIN2+ derived from a post hoc analysis of a randomised trial control arm only partly follow the same ordering: HPV16 (hazard ratio 10.44, 95% CI 6.96–15.65) and HPV33 (9.65, 95% CI 5.97–15.60) are highest, but HPV18 (3.87, 95% CI 2.38–6.30) ranks below HPV31 and HPV45 [3]. The separation between the second and third tiers is modest. In the Danish cohort the 12-year CIN3+ risks for HPV18 (19.1%), HPV33 (14.9%) and HPV31 (14.3%) overlapped substantially [4].

The genotype hierarchy carries three qualifications. The relationship between CIN3 and cancer is not uniform across genotypes: in a meta-analysis of 115,789 HPV-positive women, HPV18 and HPV45 were relatively under-represented in CIN3 compared with invasive cancer (invasive-cancer-to-CIN3 prevalence ratios 2.11 and 1.47, versus 1.08 for HPV16) [26]. That pattern is consistent with a shorter or less conspicuous precursor phase and with the predominance of these types in glandular disease. Genotype distribution also differs by region, so tiers derived in North American and European cohorts require local confirmation [26]. Finally, all of these tiers were derived before vaccinated birth cohorts entered screening, and they will require recalibration.

### 3.3. Duration and Type Concordance of Persistence

Duration modifies risk within genotype. In a population-based cohort with two visits approximately one year apart, the three-year cumulative incidence of CIN2+ was 17.0% (95% CI 12.1–22.0) among women positive for any hrHPV type at both visits. The corresponding figures were 3.4% (95% CI 0.1–6.8) for negative-then-positive, 1.2% (95% CI −0.2 to 2.5) for positive-then-negative and 0.5% (95% CI 0.1–0.9) for doubly negative. For short-term HPV16 persistence, the five-year cumulative incidence of CIN2+ reached 40.8% (95% CI 26.4–55.1) [27]. Analyses of a large longitudinal cohort indicate that duration of persistence, and not a woman’s age, drives risk once genotype is accounted for [22].

Concordance of genotype between visits carries information distinct from duration. In a Chinese cohort of women with abnormal cytology and normal cervical histology, progression was assessed after two years. Relative to women who remained hrHPV-negative, detection of the same genotype at follow-up carried an adjusted odds ratio of 22.26 (95% CI 8.98–55.17) for progression to CIN1 or worse. Detection of a different type was not significantly associated with progression (3.92, 95% CI 0.81–18.99), and only 10 of the 32 progression events were CIN2/3 [28]. Only 357 of the 1232 women (29.0%) were hrHPV-positive at entry, and persistence before entry was not documented. The study population therefore corresponds to the histological component of state 3a rather than to its full, persistence-based definition [28]. The cohort does not provide a direct estimate of prognosis for women with documented persistent hrHPV after negative histology. In the NTCC2 study, all 11 CIN3 cases reported overall occurred in women with persistent infection and none in those with exclusively new infection. Three CIN3 cases were also reported among women who remained positive on the screening HPV test but were negative on the genotyping assay. The published overall count is therefore not fully reconcilable with the subgroup distribution, which limits interpretation of the subgroup-specific findings [29]. The joint contribution of genotype and of persistence is shown in Figure 1, and the principal longitudinal studies underpinning these estimates are tabulated in Table S1.

### 3.4. Redetection, Latency and the Limits of Clearance

A negative HPV test does not necessarily indicate eradication. Redetection of the same genotype after apparent clearance has been reported in 10–20% of women, and much incident detection is unrelated to recent sexual exposure. In a prospective cohort of women aged 35–60 years, 72% of incident detections occurred in women reporting five or more lifetime partners and were not associated with a recent partner. In a separate cohort, the proportion of incident detections judged to represent probable new infection fell from 48% among women aged 30–39 years to 21% among those aged 40–50 [30]. The proposed substrate is maintenance of viral genomes at low copy number in basal keratinocytes. Whether a DNA-negative state represents virological cure or control below the detection limit of current assays is unresolved [30].

Residual risk after documented clearance is measurable. Among hrHPV-positive, cytology-negative women in a randomised screening trial whose repeat HPV test was negative, the five-year CIN3+ risk was 2.0% (4/199) against 0.2% (41/18,562) in doubly negative women [31]. Clearance reduces but does not normalise risk, which argues against returning a previously persistently positive woman immediately to the standard screening interval.

## 4. Absence of Disease Versus Failure of Detection

### 4.1. The Performance Ceiling of Colposcopy

A negative colposcopic assessment does not exclude CIN2+ with certainty, and estimates of colposcopic accuracy share a structural constraint. The colposcopic impression directs the biopsy that constitutes the reference standard, so disease in a normal-appearing cervix is under-ascertained; sensitivity may therefore be overestimated in studies using colposcopically directed biopsy as the principal reference standard. With that caveat, a meta-analysis of 49 studies and 141,355 examinations, conducted predominantly in women referred with abnormal cytology, gave a pooled sensitivity for CIN2+ of 0.745 (95% CI 0.684–0.797) with specificity 0.831 (95% CI 0.770–0.879), alongside substantial statistical heterogeneity between the included studies [32]. Performance depends heavily on biopsy practice. In a prospective study of 690 referred women, sensitivity for HSIL rose from 60.6% (95% CI 54.8–66.6) with a single biopsy to 85.6% (95% CI 80.3–90.2) with two and 95.6% (95% CI 91.3–99.2) with three. Non-targeted biopsy of normal-appearing transformation zone accounted for only 2–4% of all HSIL detected [33]. That last figure reflects a limited incremental yield in this referral population and cannot be extrapolated to all clinical settings; nor does it account for the disease that colposcopy misses. The endocervical and glandular compartments discussed below absorb only a small part of it, and much of the remainder reflects failure of the colposcopic impression itself.

In the population of interest here, measured sensitivity is lower than that benchmark. Among 372 cytology-negative, hrHPV-positive women biopsied at colposcopy in a Chinese referral centre, colposcopic impression had a sensitivity of 50.0% and a specificity of 96.9% for CIN2+ [34]. The same series reported invasive carcinoma in 2.42%, a rate that reflects a referral and not a screening population. This is a single retrospective series, and it was the only study our searches identified that measured colposcopic sensitivity directly in cytology-negative hrHPV-positive women. The estimate should be read as indicative and not as established. Among women referred through an organised programme for persistent hrHPV with normal or borderline cytology, most confirmed high-grade lesions lacked classical colposcopic features, and structured colposcopic scoring showed poor sensitivity [5]. Where the transformation zone is not fully visible, measured sensitivity was 51.2%, with specificity 96.5%, in 764 women with a type 3 transformation zone [35]. Both sensitivity estimates sit well below the pooled referral benchmark of 74.5%. Their proximity does not show that an incompletely visible transformation zone leaves colposcopic sensitivity unaffected, because the two series differ in population and biopsy protocol and more than half of the women in the first also had a type 3 transformation zone [34]. In a type 3 transformation zone, endocervical disease cannot be fully assessed colposcopically, and selective endocervical sampling, as performed in both series, may leave the reference standard incomplete [34,35].

Whether computational assistance raises this ceiling is unsettled. A meta-analysis of artificial-intelligence systems applied to colposcopic images reported diagnostic accuracy for CIN2+ broadly comparable to expert colposcopy, with wide heterogeneity in endpoints, devices and validation design [36]. A prospective multicountry evaluation of an automated visual-evaluation tool in five African settings likewise found performance dependent on image quality and on the prevalence of the target condition [37]. Both bodies of work share the verification bias described above, because the reference standard remains colposcopically directed biopsy. Neither has been evaluated in cytology-negative women with documented persistence, where the pretest probability is low and the lesions that matter are the flat and endocervical ones least represented in training sets. Artificial intelligence is therefore not yet an answer to the specific failure mode described here, although it is the most active area of methodological work bearing on it.

### 4.2. The Endocervical Compartment

When the squamocolumnar junction is not visible, the diagnostic question shifts from what can be seen to what can be sampled. Endocervical curettage detected endocervical CIN2+ in 14.4% (95% CI 10.0–20.2) of women undergoing colposcopy for abnormal cytology. Its incremental yield beyond up to four directed ectocervical biopsies was only 3.9% (95% CI 1.9–7.8) and below 5% when the colposcopic examination was normal or satisfactory [38]. Current standards accordingly recommend curettage when the squamocolumnar junction is not fully visualised, when HPV16 or HPV18 is present, and preferentially from age 40 years and regard it as unacceptable in pregnancy [39]. In a retrospective series of 690 women with a type 3 transformation zone, curettage was the only source of a CIN2+ diagnosis in 2.8% (19/690). The yield was concentrated in HPV16/18-positive women (4.5% versus 1.3% for other genotypes; *p* = 0.017; adjusted odds ratio 3.22, 95% CI 1.10–9.44), with 22.1 curettages needed to detect one additional CIN2+ in HPV16/18-positive women versus 76.0 in others [40]. That series was assembled from a colposcopy referral population in which only 16.8% had negative cytology and CIN2+ prevalence was 48.0%, so the absolute yields are not transportable to the woman without confirmed CIN2+; the genotype gradient is the transferable finding. Detection yields across these settings are collated in Table S2.

### 4.3. Glandular Disease Is Poorly Served by Morphological Assessment

Adenocarcinoma in situ and endocervical adenocarcinoma are the lesions least likely to be identified by the modalities that define a lesion as absent. In a retrospective series of 569 adenocarcinoma in situ and 498 endocervical adenocarcinoma cases, hrHPV was detected in approximately 97.7% of adenocarcinoma in situ and 90.2% of HPV-associated adenocarcinoma, whereas abnormal cytology was present in approximately 71.1% and 71.9% respectively. For HPV-independent adenocarcinoma the pattern reversed, with hrHPV detected in about 16.5% and abnormal cytology in 60.7%. Co-testing identified 72.2% of that subtype against 100% of adenocarcinoma in situ cases and 94.3% of HPV-associated adenocarcinoma [41]. These are test-positivity rates measured retrospectively in already-diagnosed cases, not prospective screening sensitivities, and they are therefore subject to verification bias. Consistent with them, approximately half of the cancers occurring after an hrHPV-positive, cytology-negative co-test in a large screening cohort were adenocarcinomas [20]. For HPV-associated glandular disease the viral test carries more diagnostic weight than the morphology; for HPV-independent adenocarcinoma neither modality performs well.

### 4.4. The Reproducibility of a Negative Histological Result

Histological interpretation is no more reproducible than cytology. Kappa values in a large reproducibility study were 0.46 (95% CI 0.44–0.48) for cytology, 0.46 (95% CI 0.43–0.49) for punch biopsy and 0.49 (95% CI 0.44–0.55) for loop excision specimens, with irreproducibility greatest for the less severe interpretations that define this clinical scenario [42]. The scope for adjudicating such a result by immunohistochemistry is narrower than is sometimes assumed. Under the Lower Anogenital Squamous Terminology criteria, p16 is recommended when the morphological differential lies between precancer and a mimic of precancer, when the interpretation under consideration is CIN2, and as an adjudication tool in professional disagreement where the differential includes CIN2 or CIN3. Routine p16 staining of biopsies interpreted as negative, CIN1 or CIN3 is explicitly recommended against [19]. The one exception relevant here is a biopsy of CIN1 or less in a specimen at high risk of missed high-grade disease, which the criteria define by the preceding cytological interpretation: HSIL, ASC-H, ASC-US with HPV16 positivity, or atypical glandular cells not otherwise specified [19]. Most women in the population of this review have negative cytology and so fall outside that definition. p16 positivity does not by itself convert a morphological CIN1 into HSIL, and immunohistochemistry cannot be used to resolve the diagnostic uncertainty created by a persistently positive viral test in a cytology-negative woman.

### 4.5. Four Explanations for the Same Clinical Picture

Four non-exclusive explanations fit a persistently positive hrHPV test without confirmed CIN2+. The first is a productive infection that has not transformed the epithelium and may still regress. The second is a latent or low-copy infection maintained in the basal compartment. The third is an occult transforming lesion within the endocervical canal, glandular in type, or too flat to be identified colposcopically. The fourth, less common, is a source in the vaginal epithelium. They differ biologically. No assay in current use separates the first two, which are in any case managed identically. Repeating the same morphological assessment without additional risk information is unlikely to distinguish between them. We identified no study quantifying the diagnostic yield of repeat colposcopy after a prior adequate negative examination in this population. That gap is the principal argument for adding molecular triage instead of repeating the morphological one.

One consideration bounds the significance of occult disease, and it concerns the endpoint, not the magnitude. Where persistently positive, cytology-negative women are examined systematically, high-grade histology is found in a minority: HSIL in 13.6% of those referred through an organised programme with normal cytology [5] and CIN2+ in 7.6% of a German colposcopy series [6]. Both are composite endpoints combining CIN2 with CIN3. Because CIN2 and CIN3 differ substantially in natural history and in clinical consequence, with half of untreated CIN2 regressing within 24 months [43], a detection rate expressed as CIN2+ or HSIL must not be read as an estimate of occult CIN3+ risk. The occult CIN3+ fraction is smaller: the German series reports CIN3+ in 12 of 237 women with normal cytology (5%) [6]. This does not diminish the importance of detecting genuine high-grade disease. It means that the composite figures overstate the risk that intensified assessment is intended to address.

## 5. Risk Stratification and Triage in Women Without Confirmed CIN2+

The options below are compared on four distinct properties, which are frequently conflated. Detection asks whether the test identifies prevalent CIN2+ or CIN3+ at the index examination. Risk stratification asks whether the result materially changes the subsequent probability of CIN3+, which is a question about reclassification, not about detection. Prognostic enrichment asks whether the marker identifies the infections that will progress and not those that will regress. Clinical utility asks whether using the test changes management, reducing colposcopy or treatment without a loss of safety. High sensitivity and specificity establish the first property alone. Of the options considered here, only extended genotyping and p16/Ki-67 dual-stain cytology have been carried through to guideline-adopted clinical utility. Methylation and viral integration have prognostic-enrichment data but no adopted management rule. E6/E7 messenger RNA has been validated as a primary screening assay, not as a triage step after documented persistence.

The evidence for these options also differs in how directly it applies to the women considered here, and three levels can be distinguished. A minority of estimates were obtained in women with documented persistence after a previous assessment, such as the retesting data in Section 5.1 and the referral cohort in Section 5.2. Most were derived from a single hrHPV-positive result in screening or co-testing cohorts, and the markers have been evaluated chiefly for their ability to stratify HPV-positive women [13]. Applied to women with documented persistence and a previous negative or non-high-grade assessment, those estimates are transported rather than derived. Sensitivity and specificity depend on the spectrum of disease and on how completely outcomes were verified, and predictive values depend in addition on prevalence and diagnostic history. Persistent positivity raises the prior probability of high-grade disease (Section 3), whereas a negative colposcopy or biopsy removes much of the detectable disease and may leave lesions that are harder to sample (Section 4). Positive and negative predictive values reported at an initial HPV-positive screen should therefore not be assumed to hold unchanged after persistent positivity and a negative colposcopy or biopsy. This concerns the directness of the evidence, not the validity of the markers themselves; the question here is how they allocate diagnostic intensity within this clinical problem.

### 5.1. Repeat Testing: Necessary but Not Sufficient

Repeating the HPV test is the default triage in most programmes, and its value depends on the interval chosen (Section 2.1). Repeat testing also cannot, by itself, distinguish a persisting infection from a newly acquired one. Extended genotyping applied at a median of 13.5 months after a baseline positive test reclassified 68 (11.1%) of 613 women who remained positive at retesting and had available genotyping results as having exclusively new infections. The investigators estimated that this could reduce total colposcopy referrals by about 7% [29].

### 5.2. Cytology

Cytology retains discriminatory value despite its limitations. Among women referred for persistent hrHPV, HSIL was confirmed in 42 of 156 with atypical squamous cells of undetermined significance compared with 46 of 337 with normal cytology [5]. Its weaknesses are equally clear. The immediate CIN3+ risk associated with hrHPV positivity and negative cytology is only 2.1%, below the threshold at which colposcopy is recommended in risk-based frameworks [44]. A repeat hrHPV-positive, cytology-negative result carries a higher five-year CIN3+ risk than the same result at baseline (7.4% versus 4.5%) [20], and reproducibility of low-grade interpretations is modest [42].

### 5.3. Extended Genotyping

Genotyping beyond HPV16 and HPV18 discriminates CIN3+ risk independently of cytology, although assays and populations differ sufficiently that pooled estimates of accuracy are not available. In an organised European programme, extended genotyping identified a group of genotypes whose 24-month CIN3+ risk among women with negative cytology was as low as 0.4% (a figure that included women negative on the genotyping assay; 0.5% when they were excluded), which the investigators judged low enough for retesting after 3 years [45]. That judgement rests on a 24-month estimate and does not map directly onto the five-year thresholds used in North American guidance. The risk gradient among cytology-negative women is quantified in the evidence supporting the current enduring guidelines. Immediate and three-year CIN3+ risks were 8.4% and 11.5% for HPV16, 4.0% and 8.5% for HPV18, 1.8% and 2.9% for the intermediate-risk types, and 0.4% and 0.9% for HPV56/59/66 [46]. These estimates derive from a different cohort and a later period than those underpinning the 2019 risk tables, which is why the HPV18 figure quoted in Section 6.1 differs. Across those immediate-risk estimates, a single hrHPV-positive, cytology-negative result spans an approximately twenty-fold range of absolute risk, from 0.4% to 8.4%. The estimates were modelled from one co-test result, irrespective of earlier results, and not from documented persistence; the guideline panel judged type-specific data for repeated rounds of testing insufficient to support risk-based recommendations [46]. In May 2026 the World Health Organization published a dedicated guideline on the use of HPV DNA genotyping [47]. It formalises a comparable hierarchy for the 12 carcinogenic genotypes: group 1a (HPV16), group 1b (HPV18 and HPV45), group 1c (HPV31, 33, 35, 52 and 58) and group 1d (HPV39, 51, 56 and 59). Extended genotyping is thereby established as a molecular triage tool in global guidance and no longer only in North American practice. The grouping corresponds closely to the risk tiers derived from cohort data in Section 3.2, with the difference that HPV66 and HPV68 fall outside the 12 types the WHO classifies as carcinogenic. The WHO genotype groups should nevertheless be distinguished from the risk categories used in the US enduring guidelines; although both frameworks stratify management by genotype-associated risk, they were developed independently and do not classify all genotypes identically.

### 5.4. p16/Ki-67 Dual-Stain Cytology

Dual-stain cytology has been evaluated prospectively in several large screening cohorts. In a cohort of 1549 hrHPV-positive women, the five-year risk of CIN2+ was 31.0% (95% CI 27.2–35.3) after a positive dual stain versus 25.0% (95% CI 21.7–28.7) after abnormal cytology. After a negative result it was 8.5% (95% CI 6.5–11.1) for dual stain versus 12.3% (95% CI 9.8–15.4) for cytology [48]. In a programme-based evaluation of 3225 hrHPV-positive women, dual-stain triage yielded more CIN3+ per test-positive result than cytology (12.0% versus 10.3%; *p* = 0.005) while requiring about one-third fewer colposcopies [49]. In a prospective US screening study of 4927 hrHPV-positive women, dual-stain sensitivity for CIN3+ was 91.9% (95% CI 86.1–95.4) in HPV16/18-positive women and 86.0% (95% CI 77.5–91.6) in those positive for other genotypes. The CIN2+ risk after a negative dual stain was 3.6% (95% CI 2.9–4.4), compared with 7.4% (95% CI 6.4–8.5) after negative genotyping-plus-cytology triage and 7.5% (95% CI 6.7–8.4) after negative cytology triage (both *p* < 0.0001) [50]. Current consensus recommendations direct dual-stain-negative women to one-year follow-up, the immediate and three-year CIN3+ risks after a negative dual stain being 0.75% and 1.5% where genotyping is not available. Immediate colposcopy is retained for HPV16 and HPV18 irrespective of the dual-stain result [51]. The same recommendations address follow-up after abnormal results, colposcopy and treatment, but the supporting dual-stain data in these settings were limited [51]. Dual staining also has signal where colposcopy does not. In 101 women with a type 3 transformation zone scheduled for excision, dual-stained cytology had a positive predictive value of 76.1% against a CIN2+ prevalence of 35.9% in the low-grade referral group [52]. In that group, no CIN2+ was found in women with a negative dual stain, but the numbers are small.

### 5.5. Host and Viral DNA Methylation

Methylation markers behave differently from cytology and dual stain. They are less sensitive for prevalent CIN2/3 but preferentially identify advanced and invasive disease and can be measured on self-collected samples. At a fixed specificity of 70%, pooled sensitivity in the studies of a 43-study meta-analysis that allowed this standardisation was 68.6% (95% CI 62.9–73.8) for CIN2+ and 71.1% (95% CI 65.7–76.0) for CIN3+ [53]. In a European multicentre validation of the FAM19A4/miR124-2 assay on 2384 hrHPV-positive samples, sensitivity was 95% (19/20) for cancer and 77% (95% CI 71–82) for CIN3, with a negative predictive value for cancer of 99.9% (95% CI 99.6–99.99) [54]. Long-term data are the strongest argument for these markers. In a post hoc analysis of a randomised screening trial with 14 years of follow-up, within-round sensitivity for cervical cancer was 100% (11/11). The 14-year cumulative cancer incidence after a negative methylation test was 1.7% (95% CI 0.66–3.0) compared with 2.4% (95% CI 1.4–3.6) after negative cytology (risk difference 0.71%, 95% CI 0.16–1.4) [55]. A multimarker classifier discriminated women who would later develop CIN3 or invasive cancer from hrHPV-positive controls using archived specimens taken on average five years before diagnosis, independently of baseline cytology. Sensitivity for prevalent CIN3 was 93% versus 61% for HPV16/18 genotyping (*p* = 0.01), at the cost of lower specificity (50% versus 66%) [56]. Methylation can also be applied to self-collected samples, on which an ASCL1/LHX8 panel detected CIN3+ with sensitivity 73.3% and specificity 61.1% [57]. We identified no programme-level guideline that has adopted methylation as a routine triage step, and thresholds are not standardised across assays.

### 5.6. Markers Not Yet Ready for Practice

Testing for E6/E7 messenger RNA offers greater specificity than DNA testing without a demonstrable loss of sensitivity. In a head-to-head screening comparison of 9451 women, no significant sensitivity difference for CIN2+ or CIN3+ was observed, while specificity and positive predictive value were significantly higher for the mRNA assay (both *p* < 0.001) [58]. Its role as a triage step after documented persistence has not been established. Detection of viral integration achieved a specificity for CIN3+ of 94.5% (95% CI 93.3–95.8) against 63.8% (95% CI 61.2–66.4) for cytology at equivalent sensitivity (70.5%). Integration-negative women, 90.1% of the cohort, had an immediate CIN3+ risk of 2.2%, and one-year progression was 12.0% in integration-positive versus 2.1% in integration-negative women (odds ratio 5.6, 95% CI 2.6–11.9) [59]. This is a single-cohort result requiring external validation, and no standardised assay or reporting threshold yet exists. The comparative characteristics, validation status and current guideline position of each triage option are summarised in Table 1.

## 6. Guideline Recommendations and the Anatomy of Their Divergence

### 6.1. Two Different Logics

Contemporary guidance follows one of two structures. Risk-based frameworks assign management according to the estimated absolute probability of CIN3+, using explicit numerical thresholds. Expedited treatment is preferred at an immediate CIN3+ risk of 60% or greater and is acceptable between 25% and 60%, while colposcopy is recommended at 4.0% or greater. Below that, one-year repeat testing applies when the five-year risk is 0.55% or greater, three-year repeat testing between 0.15% and 0.55%, and return to routine five-yearly screening below 0.15% [8,44]. Because hrHPV positivity with negative cytology carries an immediate CIN3+ risk of 2.1%, it falls below the colposcopy threshold and generates a recommendation for surveillance at one year [8].

Where the result persists, the framework moves to colposcopy at the second occurrence. The guideline groups hrHPV-positive, cytology-negative results occurring at two consecutive annual visits with the low-grade results whose immediate CIN3+ risk sits just above the 4.0% threshold [8]. That risk lies well below the 25% threshold at which treatment becomes an option, so colposcopy is the recommended management [44]. A correction to the guideline, carried forward in the 2024 consolidated update, addresses cases in which recommended colposcopy was not performed or no exact risk estimate is available after two consecutive HPV-positive results [8,63]. It does not modify the rule for two consecutive hrHPV-positive, cytology-negative results. For low-grade results, one-year surveillance remains the recommendation after an examination showing less than CIN2 within the preceding year; after ASC-H or HSIL cytology, observation depends on full visualisation of the squamocolumnar junction and on endocervical sampling findings, and diagnostic excision is also acceptable after HSIL [8]. Within this framework, a documented negative hrHPV-based screening result in the preceding round lowers the estimated risk associated with a subsequent abnormal result, whereas a previous negative cytology alone does not [8,44].

The framework is frequently misread in two respects. The thresholds are not applied mechanically. Colposcopy is recommended for HPV18-positive, cytology-negative women despite a prevalent CIN3+ risk of 3.0% in the risk estimates underpinning the 2019 guidelines, below the 4.0% threshold, because of a disproportionate cancer risk (0.2% immediately and 0.56% at five years) [8]. The treatment thresholds also sit roughly twelve- to thirty-fold above the risk conferred by hrHPV positivity with negative cytology, which is why no recommendation for treating persistent infection without histological confirmation exists here. The enduring-guideline process has extended the same logic to extended genotyping and dual-stain triage without altering the thresholds [46,51].

Algorithmic frameworks, used by most organised European and Commonwealth programmes, reach broadly similar destinations by categorical rules. The European position is currently in transition. The 2015 supplements to the European quality-assurance guidelines remain the most recent pan-European statement addressing triage of hrHPV-positive women in detail. They hold that direct referral to colposcopy is not recommended, either for hrHPV-positive women in general or for those with negative triage cytology, who should instead be retested after at least 6–12 months [64]. That document predates the evidence on extended genotyping and dual staining reviewed above and is cited here as legacy guidance, not as the current European position. The European Commission Initiative on Cervical Cancer released its first set of recommendations in July 2025. They recommend HPV detection as the primary screening method for people with a cervix aged 30–64 years, with a conditional recommendation from age 25 [65]. Final recommendations on triage of HPV-positive women, genotyping, surveillance and referral were not publicly available by the closing date of this review. At national level, the direction of travel is visible: the 2025 Italian multisociety recommendations, developed with Grading of Recommendations Assessment, Development and Evaluation (GRADE) methodology, issued a conditional recommendation in favour of combining extended genotyping with a morphological marker, either cytology or p16/Ki-67, in preference to cytology alone for triage of HPV-positive women [66].

### 6.2. How the Programmes Actually Differ

Comparison of the operative rules, summarised in Table 2, shows that the substantive disagreement concerns how long pure viral persistence with normal cytology may be observed before the cervix is inspected and whether genotype should shorten that interval. The English programme refers to colposcopy on the third consecutive hrHPV-positive result, irrespective of cytology, and genotyping is not used [9]. The Australian guidelines were revised in January 2025, with effect from April 2025. That programme refers HPV16/18-positive women to colposcopy immediately and repeats testing at 12 months for other oncogenic genotypes with negative or low-grade cytology, referring where a non-16/18 genotype persists across three consecutive annual tests. Earlier referral applies in defined higher-risk groups [60]. The English and Australian pathways therefore now converge on the third consecutive positive result. The Dutch programme repeats cytology at 12 months and, where cytology remains normal, returns the woman to screening instead of to colposcopy, so that pure viral persistence may never generate a colposcopic examination at all [61]. Canadian guidance refers HPV16- or HPV18-positive women to colposcopy regardless of cytology [10]. After a colposcopy without histological HSIL, women referred with low-grade cytology are discharged to HPV-based screening at 12 months; for women referred with high-grade cytology, annual colposcopic review continues until two consecutive negative HPV tests are obtained or histological HSIL is identified [10].

### 6.3. The World Health Organization Position

The largest divergence is not among high-income programmes but between them and the World Health Organization, and it follows from a difference in purpose, not in evidence. WHO guidance is written for national programmes operating under explicit constraints of follow-up and treatment capacity, and its recommendations are population-level strategies, not rules for managing an individual woman after a negative diagnostic assessment. For the general population, the 2021 second edition suggests either a screen-and-treat approach, in which women testing positive for HPV DNA are treated, or a screen, triage and treat approach. Both are conditional recommendations based on moderate-certainty evidence [11]. For women living with HIV, screen, triage and treat is suggested in preference to screen-and-treat [11]. A 2024 update added dual-stain cytology as a permitted triage option [62]. Retesting intervals for these pathways are summarised in Table 2.

A dedicated guideline on HPV DNA genotyping, published in May 2026, changes the structure of that guidance without altering the recommendation that HPV DNA testing is the preferred primary screening test [47]. It examines no, limited and extended genotyping as instruments of risk stratification and molecular triage and conditions its recommendations on programme follow-up capacity. In settings with high follow-up capacity, defined as 60% or greater, and sufficient ablative treatment capacity, the WHO suggests HPV DNA testing with extended or limited genotyping in preference either to treating all HPV-positive women or to triaging all of them with additional tests. Among the extended-genotyping algorithms, ablative treatment of eligible women in groups 1a, 1b and 1c, with triage of group 1d, is preferred over more restrictive alternatives in which fewer groups are treated [47]. The modelling underpinning that preference found larger reductions in cervical cancer where treatment was extended to more of the carcinogenic genotypes. Separate recommendations apply to settings of lower follow-up capacity. Where an additional triage test is used, the guideline conditionally suggests visual inspection with acetic acid or colposcopic impression, without histological confirmation, in preference to cytology or dual-stain cytology, regardless of follow-up capacity [47]. Implementation guidance issued in July 2026 supports countries in operationalising HPV-based screening but adds no clinical recommendations [67].

Two features bear directly on the subject of this review. Under the preferred algorithm for settings with adequate follow-up and treatment capacity, genotype determines not only the intensity of triage but, for eight of the twelve carcinogenic genotypes, whether a woman may be treated without preceding morphology-based diagnostic triage or histological confirmation. The genotype hierarchy that risk-based frameworks use to allocate colposcopy is used here to allocate ablation. The recommendations are nevertheless addressed primarily to the general population entering screening, not to the woman with documented type-specific persistence who has already undergone an adequate and negative cervical assessment.

Among the guidance compared in this review, and apart from the acceptance of excision or ablation for CIN1 persisting for at least two years in the United States guidelines [8], the WHO is the only source under which a woman with hrHPV and no confirmed CIN2+ may receive ablative treatment, whether through screen-and-treat or through the genotype-directed algorithm above. Visual assessment of the cervix is nevertheless still required before ablative treatment to establish eligibility, to select the treatment modality and to exclude suspected cancer. Both are conditional, population-level strategies for programmes in which many screen-positive women never complete the diagnostic pathway, not claims that treating infection is clinically superior to assessing it. The 2021 document also notes that ablative treatment is unsuitable when the transformation zone is not visible, which excludes much of the older population in whom persistence is most often detected [11].

### 6.4. Screening Exit

Whether a persistently hrHPV-positive woman may leave the programme is unresolved, and the guidance itself is not in conflict so much as incomplete. American Cancer Society guidance requires negative HPV-based tests at defined ages before discontinuation, so a woman who remains hrHPV-positive does not qualify for exit [68], and the WHO suggests stopping after age 50 following two consecutive negative results [11]. Both therefore condition exit on a negative result and not on age alone. For a woman who never achieves one, the American Cancer Society defers surveillance decisions to the American Society for Colposcopy and Cervical Pathology guidelines [68], under which discontinuing surveillance is unacceptable while she remains in reasonably good health and testing is feasible [8]; WHO guidance does not specify how long surveillance should continue. Long-term cohort data support caution. In two Manchester screening cohorts, the 15-year cumulative CIN3 risk after a positive hrHPV test was 11.7% and 12.0%, rising to 18.2% and 23.9% for HPV16. Invasive cancer risk continued to increase for 30 years after a single positive test, reaching 2.5% (95% CI 1.3–4.5) in the older cohort, and the authors concluded that discharging women while they remain HPV-positive may not be safe [69].

## 7. Interventions Directed at the Infection, and the Harms of Intervening

Much of the earlier interventional evidence in this field was generated in women with histologically confirmed CIN, whereas recent trials have begun to evaluate interventions directly in women with persistent hrHPV without confirmed high-grade disease. The older lesion-directed literature is retained here because the harms it documents set the standard against which any intervention in the absence of confirmed high-grade disease must be judged. Throughout this section, randomised and non-randomised evidence, populations with and without CIN1, and virological, histological and clinically meaningful endpoints are distinguished explicitly, because conflating these categories can overstate the applicability of individual findings.

### 7.1. Prophylactic Vaccination Does Not Clear an Established Infection

The only randomised test identified in this review of whether prophylactic vaccination clears an established infection was a community-based trial in 2189 HPV-DNA-positive women aged 18–25. They were randomised to bivalent HPV16/18 vaccine or a control vaccine. Vaccine efficacy for clearance of HPV16/18 was 2.5% (95% CI −9.8 to 13.5) at six months and −2.0% (95% CI −24.3 to 16.3) at 12 months, with no effect in any stratum [70]. Extended analysis of the same trial found no increase in clearance of infections present at vaccination and no significant reduction in lesions arising from them, indicating that any post-treatment benefit is attributable to prevention of new infection and not of existing infection [71]. These null estimates have wide confidence intervals, and the trial population was younger than women who typically present with documented persistence. The absence of a therapeutic effect on an established infection does not negate the prophylactic benefit of vaccination against genotypes not yet acquired or its appropriateness for women eligible under age-based or risk-based recommendations.

The literature on vaccination after excisional treatment is often invoked in support of vaccinating persistently infected women, and the analogy does not hold. A meta-analysis of 17 studies and 33,181 women reported a pooled relative risk of recurrent CIN2+ of 0.38 (95% CI 0.29–0.51; I^2^ = 58.6%), but the estimate is dominated by non-randomised cohorts and the randomised trials within it show an attenuated effect (meta-regression relative risk approximately 0.55) [72]. The distinction that matters is one of mechanism: benefit observed after excisional treatment of CIN cannot be extrapolated to therapeutic clearance of an established infection in a woman without confirmed CIN2+. Vaccination should not be represented to her as treatment of the infection she already has.

### 7.2. Therapeutic Vaccines and Lesion-Directed Topical Agents: Evidence from Adjacent Populations

The strongest efficacy data for the lesion-directed therapeutic vaccines and topical agents discussed below were generated predominantly in women with histologically confirmed CIN2/3, where the treatment target was a lesion and not isolated viral persistence. A DNA vaccine targeting HPV16/18 E6 and E7 achieved per-protocol regression in 49.5% versus 30.6% of placebo recipients in a phase 2b trial (difference 19.0 percentage points, 95% CI 1.4–36.6; *p* = 0.034), without published phase 3 confirmation [73]. Topical imiquimod produced regression to CIN1 or less in 61% versus 23% (*p* = 0.001; per-protocol analysis of 78 of 90 enrolled women) in women with HSIL [74] and intravaginal 5-fluorouracil regression in 93% versus 56% of those with a six-month biopsy (intention-to-treat relative risk 1.62, 95% CI 1.10–2.56) in 60 women with CIN2 [75]. One therapeutic vaccine trial was identified in the population relevant to this review: a phase I study of 47 HPV16/18-positive women with normal cytology, assessing safety and immunogenicity and reporting only exploratory virological comparisons between arms [76].

### 7.3. Agents Directed at the Infection Itself

The older evidence closest to this population is negative. A randomised, double-blind, placebo-controlled trial of oral green tea catechins in 98 women with persistent hrHPV and CIN1 found complete response in 17.1% versus 14.6% of placebo recipients, with no effect on viral clearance [77]. This population corresponds to state 3b only. A randomised placebo-controlled trial of oral lactobacilli in 121 hrHPV-positive women found clearance of 58.1% versus 54.2% (log-rank *p* = 0.741) [78]. Participants were selected by negative cytology, without histological verification; colposcopic biopsy was reserved for ASC-US or worse. A carrageenan-based gel reduced acquisition of new infection in a phase IIB randomised trial but did not significantly accelerate clearance of established infections [79]. In a substudy of HPV42 and HPV51 infections, gel use did not significantly reduce viral load [80]. That trial did not select participants by cervical histology. The only randomised trial of a *Coriolus-versicolor*-based vaginal gel was open-label against watchful waiting. Its primary endpoint, normal cytology with concordant colposcopy at six months, favoured the gel (84.9% versus 64.5%; *p* = 0.031), although colposcopy was assessed without blinding. HPV clearance, a secondary endpoint that included partial clearance, did not differ significantly in the pooled comparison (62.5% versus 40.0% among hrHPV-positive women; *p* = 0.076). It did differ for one of the two dosing schedules [81]. Its participants had low-grade cytology with concordant colposcopic findings, and no biopsies were performed [81]. An independent appraisal found the remaining literature on this product to be uncontrolled, retrospective or industry-sponsored [82]. None of these four studies therefore provides evidence specific to state 3a.

Two randomised trials published in 2026 have changed the shape of this evidence, and they point in opposite directions. In a three-arm trial at a single Chinese centre, 842 women were screened and 120 women with persistent same-type hrHPV were randomised 1:1:1 to the PHPV^®^ fusion-factor vaginal gel, intravaginal interferon, or no treatment; 110 entered the modified intention-to-treat analysis. Women with cervical cancer or high-grade lesions were excluded, and HPV testing, cytology and colposcopic biopsy were obtained within one month before enrolment. The trial therefore represents a histologically assessed population without confirmed CIN2+, corresponding to state 3. Of the 120 women randomised, 50 (41.7%) had negative or inflammatory histology (state 3a) and 70 (58.3%) had CIN1 (state 3b) [83]. At six months, hrHPV clearance was 66.7% with the gel, 45.9% with interferon and 20.6% with no treatment. After adjustment the gel remained superior to no treatment (relative risk 2.81, 95% CI 1.37–5.79; *p* = 0.005), whereas interferon did not (2.08, 95% CI 0.98–4.44; *p* = 0.058), and no serious adverse events were reported [83]. The trial was open-label against an untreated control, single-centre and small, the product was supplied by its manufacturer, and the endpoint was virological at six months. It does not establish durable clearance or any reduction in CIN3+ risk. Because the accessible published results do not provide clearance estimates stratified by baseline histology, the trial also cannot establish whether any effect differs between states 3a and 3b.

The second trial reached the opposite conclusion for a different product. A single-centre, open-label, parallel-group randomised trial allocated 242 women with polymerase chain reaction-confirmed hrHPV and no indication for immediate treatment 1:1 to three months of daily intravaginal silicon-dioxide-based gel or to observation alone. All women underwent baseline colposcopy, with biopsy directed by colposcopic and clinical findings; histology showed CIN1 or LSIL in five women (state 3b) and CIN2 or HSIL in one. Absence of detectable hrHPV DNA at three months was 18% with the gel and 21% with observation. Patient-reported adverse events were more frequent with the gel (32% versus 2%) and satisfaction was lower. The investigators declared no conflicts of interest and concluded that the trial did not reproduce the large short-term virological benefit suggested by earlier product-related studies [84]. The enrolled population was not entirely restricted to the phenotype reviewed here, but this does not materially affect the negative result. The randomised comparison was not reported by baseline histology, so the trial provides no estimate of effect specific to state 3a or state 3b.

Non-randomised evidence should be read separately. In a single-centre prospective cohort of 237 women with persistent HPV for at least one year and histologically confirmed CIN1, 106 chose thermal ablation plus a three-month course of an HPV-specific IgY vaginal gel and 131 chose watchful waiting. At 12 months, lesion regression was 65.1% versus 35.9% and HPV clearance 67.9% versus 35.1%, both *p* < 0.001, while progression to CIN2+ did not differ significantly (4.7% versus 8.4%; *p* = 0.267) [85]. This population falls within the broader phenotype of persistent HPV without confirmed CIN2+, but it represents state 3b only, not state 3a or women with no visible lesion. Its lesion-regression endpoint, assessed by combined cytology and histology, has no counterpart in state 3a. Treatment was self-selected and not randomised, so confounding by indication cannot be excluded, and the incremental contribution of the vaginal gel over thermal ablation alone cannot be separated within this design.

The closest synthesis is the systematic review and meta-analysis of alternatives to surveillance outlined in Section 1. It concluded that some interventions, among them vaginal gels, photodynamic therapy and certain oral agents, may bring forward resolution of persistent hrHPV and regression of low-grade CIN compared with natural clearance [12]. Because its eligible population combined women with persistent hrHPV and women with low-grade disease up to CIN1, its pooled signal is not specific to any one of the three clinical states defined here. The constituent studies are also small, heterogeneous and variably blinded.

Taken together, controlled evidence for treating persistent hrHPV without confirmed CIN2+ is sparse and conflicting, not absent. Two randomised trials of vaginal products published within five months of one another reached opposite short-term virological conclusions; the positive one was open-label, small, single-centre and manufacturer-supplied, the negative one independent and larger. No trial has followed women to CIN3+ or cervical cancer. None of the controlled studies reviewed here reported outcomes separately for states 3a and 3b in their accessible published results. Short-term loss of HPV detectability is not the same as prevention of precancer, and current evidence does not support routine treatment given solely to eradicate the virus. Regression of CIN1, an endpoint available only in state 3b, is likewise not equivalent to prevention of CIN2+, CIN3+ or invasive cancer. The evidence for each class is set out in Table S3. On the available evidence, prescribing commercially marketed vaginal products solely to achieve hrHPV clearance in women without confirmed CIN2+ cannot be recommended in routine practice, whether off-label or within a product’s marketed claims. Commercial availability does not in itself establish efficacy, and short-term virological effects have not been shown to translate into prevention of CIN3+ or cervical cancer. The conclusion reflects an absence of demonstrated clinical benefit, not proof of ineffectiveness, and does not extend to prophylactic HPV vaccination (Section 7.1).

### 7.4. Excision Without a Histological Lesion

None of the guidelines compared in Table 2 recommends routine excision or ablation for persistent hrHPV in the absence of CIN2+ outside the WHO’s population-level screen-and-treat and genotype-directed strategies [8,10,11,47]; Table 2 notes two options for defined circumstances: treatment of CIN1 persisting for at least two years [8] and excision considered after a repeat inadequate colposcopy [9]. Excision performed for a different indication, inability to sample the cervix adequately, has a real yield. Among 89 highly selected women undergoing excision for HPV persistence, repeated cytological abnormality, a type 3 transformation zone or cervical stenosis, histological abnormality was found in 53.9%, CIN2+ in 25.8%, CIN3 in 10.1%, and two early invasive carcinomas (2.3%) [86]. It does not, however, reliably eradicate the infection. Among 100 women aged 45 years or older with a type 3 transformation zone, only 70% had become HPV-negative by the end of a median 2.9 years after excision, and older women were less likely to clear (hazard ratio 0.49, 95% CI 0.28–0.87) [87]. Excision here is a diagnostic manoeuvre for the woman whose cervix cannot be assessed, not a treatment for viral persistence.

### 7.5. Quantified Harms of Excisional Treatment

In women of reproductive age, local treatment carries a depth-dependent obstetric penalty. In a meta-analysis of 71 observational studies and more than 6.3 million participants, preterm birth before 37 weeks occurred in 10.7% of treated versus 5.4% of untreated women (relative risk 1.78, 95% CI 1.60–1.98), graded by cone depth. The relative risk was 1.54 (95% CI 1.09–2.18) for the shallowest excisions and 4.91 (95% CI 2.06–11.68) at depths of 20 mm or more. The same analysis found that untreated women with CIN already had a modestly higher risk than the general population (5.9% versus 5.6%; relative risk 1.24, 95% CI 1.14–1.35), so part of the association reflects shared risk factors and not the procedure alone [88]. A network meta-analysis found relative odds of preterm birth versus no treatment of 2.27 (95% CI 1.70–3.02) for cold-knife conisation, 1.37 (95% CI 1.16–1.62) for large-loop excision and 1.05 (95% CI 0.78–1.41) for laser ablation, while cautioning that the evidence was predominantly observational [89]. Against this, half of untreated CIN2 regresses within 24 months (50%, 95% CI 43–57) [43], which is why active surveillance is now formally endorsed for selected women with CIN2 [90].

The obstetric argument does not apply to the older women in whom diagnostic excision is most often considered. For them the relevant costs are bleeding and discharge, cervical stenosis with subsequent inability to sample, incomplete diagnostic yield, and persistence of the infection in roughly 30% despite the procedure [87]. Investigation carries costs at any age. A positive HPV result with normal cytology is associated with increased short-term anxiety compared with a normal screening result (mean difference on the State-Trait Anxiety Inventory 6.33, 95% CI 1.31–11.35) and with increased psychological distress (standardised mean difference 0.68, 95% CI 0.32–1.03) [91]. Neither difference persists long-term. Pain, bleeding and discharge follow biopsy in 53%, 79% and 46% of women and excision in 67%, 87% and 63% [92]. Prolonged surveillance pathways also leak, with colposcopy non-attendance of 28.9% in a population-based cohort, although hrHPV-positive women there were more likely to attend than women referred for other abnormal results (adjusted odds ratio for attendance 3.04, 95% CI 1.49–7.22) [93].

Deferral, where risk permits, appears compatible with safety over the short term. Among 454 women with non-16/18 hrHPV and normal cytology, CIN2+ was found in 10 of the 142 with an abnormal colposcopic impression who underwent immediate biopsy (7.0%). Of the 312 women with a normal colposcopy who were followed instead of biopsied, 276 completed 12-month follow-up and no CIN2+ was detected (*p* = 0.033 for the between-group comparison) [94]. Allocation was not randomised; only 29 of the 276 women were biopsied at follow-up, so ascertainment in the deferred arm was incomplete; and with zero observed events the one-sided 95% upper bound is about 1.1%. The finding is therefore reassuring, but not confirmatory.

## 8. Populations and Contexts in Which the Balance Shifts

### 8.1. Women Living with HIV

Women living with HIV have a six-fold higher risk of cervical cancer than women without HIV (relative risk 6.07, 95% CI 4.40–8.37), the burden concentrated in southern and eastern Africa [95]. Guidance reflects this by suggesting triage in preference to untriaged screen-and-treat and shortening the interval between a negative triage result and retesting from 24 to 12 months [11]. Existing guidance therefore already specifies a shortened interval for this group. No trial defining the optimal interval was identified; the recommendation rests on the elevated cancer risk and not on comparative data. These observations are specific to women living with HIV. We did not identify evidence of comparable quality for solid-organ transplant recipients, recipients of haematopoietic stem-cell transplantation or women on chronic immunosuppressive therapy, and the surveillance interval recommended here should not be generalised to those groups.

### 8.2. Screening History

Screening history is a formal modifier of risk within the risk-based framework. A documented hrHPV-negative screen in the preceding round substantially lowers the risk associated with a subsequent abnormal result, whereas a previous negative cytology alone does not [8,44]. A woman whose infection is newly detected against a documented negative background therefore occupies a different risk position from one whose positive results extend across successive rounds, even when the current results are identical. It is also the variable most often missing, since screening histories are frequently incomplete when women move between programmes.

### 8.3. Postmenopausal Women and the Type 3 Transformation Zone

Older women present the inverse problem: lower prevalence of infection, lower yield of high-grade disease, and much poorer assessability. Pooled hrHPV prevalence among women aged 50 or older with normal cytology is 6.45% (95% CI 5.45–7.53) [96]. Detection yield also falls with age, from 78 of 345 women under 50 to 10 of 148 aged 50 or older in a persistent-HPV referral cohort [5]. A large United Kingdom cohort of women referred with persistent hrHPV and negative cytology likewise found that CIN2+ detection declined with age [97]. Yet in a multicentre series of 211 women aged 65 years or older with persistent hrHPV and negative cytology, CIN2+ was found in 20 of the 182 with a biopsy or excision specimen. Half of the CIN2+ cases were HPV16-positive. A type 3 transformation zone was present in 185 of the 211 women (87.7%), and sampling during colposcopy was not possible in 35 (16.6%). The investigators concluded that colposcopy is of limited value for identifying who needs treatment in this age group [7]. Where the transformation zone cannot be assessed, the decision is between accepting diagnostic uncertainty and performing an excision that is diagnostic and not therapeutic and that leaves 30% of women still HPV-positive nearly three years later [87]. Genotype and dual staining retain discriminatory value in women whose transformation zone is not visible, which repeat colposcopy by definition does not [40,52]. Both supporting series were assembled from colposcopy referral populations with high lesion prevalence, so the direction of the effect transfers to the woman without confirmed CIN2+ more securely than its magnitude.

Persistent hrHPV positivity should be considered explicitly when programme-specific screening-exit criteria are applied, and age alone should not be assumed to normalise residual risk. The distinction between sources matters here. The cohort evidence summarised in Section 6.4 describes long-term risk after a positive test; the exit criteria themselves are set by each programme, and both the American Cancer Society and WHO already condition discontinuation on a negative result. Neither the cohort data nor the guidance provides an estimate of residual risk for a woman who remains positive at the age at which exit would otherwise be considered.

### 8.4. Disease Outside the Cervix

A persistently positive sample is not always a statement about the cervix alone. After hysterectomy, high-grade vaginal intraepithelial neoplasia occurred in 8 of 170 women (4.7%), all of whom had persistent HPV infection [98]. Eight events cannot support a change in practice, and the observation establishes only that vaginal disease arises in persistently infected women and not the converse. These data support awareness of the vaginal compartment when the cervix appears normal or is absent, particularly after hysterectomy, but are insufficient to establish a distinct surveillance strategy. The finding should be treated as hypothesis-generating.

## 9. Synthesis, Unresolved Questions and Research Priorities

### 9.1. What the Evidence Collectively Supports

Four conclusions are supported by convergent evidence across the sources reviewed. Consistent with the narrative design, no formal certainty-grading framework was applied. The first is that persistent hrHPV positivity without confirmed CIN2+ is a quantifiable risk state and not a lesion diagnosis and that it covers three clinical situations of unequal informational value. Genotype and the duration of type-specific persistence jointly determine where a woman sits within it, and genotype alone spans an approximately twenty-fold range of immediate CIN3+ risk in hrHPV-positive screening populations [22,27,46]. The second is that a negative or non-high-grade assessment reduces but does not eliminate the probability of prevalent high-grade disease. Pooled colposcopic sensitivity for CIN2+ was 74.5% across 49 heterogeneous referral studies [32]; one selected retrospective series of cytology-negative hrHPV-positive women reported 50.0% [34], and a further retrospective series restricted to a type 3 transformation zone reported 51.2% [35]. The two lower figures come from single centres in selected populations and are not interchangeable with the pooled estimate, but they indicate that colposcopic performance in this setting should not be assumed to match the referral benchmark. Selected referral cohorts have nonetheless reported HSIL/CIN2+ detection yields of 13.6% and 7.6% among persistently positive women with normal cytology examined systematically [5,6]; both are composite, selection-dependent endpoints, and neither estimates occult CIN3+ risk. The third is that molecular triage genuinely reclassifies risk. Dual-stain cytology and extended genotyping are embedded in consensus guidance [46,51], and extended genotyping now carries a formal genotype hierarchy in global guidance as well [47], although most of the supporting validation was performed in HPV-positive screening populations rather than after documented persistence [46,51]. Long-term cancer incidence after a negative methylation result was lower than after negative cytology in one post hoc trial analysis [55], and no programme-level guideline identified here has adopted methylation into a triage algorithm. The fourth is that controlled evidence for treating the infection itself is sparse and conflicting rather than absent: recent randomised trials of vaginal interventions report discordant short-term virological results, none demonstrates durable clearance or a reduction in CIN3+ risk, and the harms of intervening are quantified and, for excision in women of reproductive age, obstetrically significant [83,84,88].

Much of the disagreement between guidelines follows from these facts and not from disputed evidence, and it is substantive rather than apparent. Programmes differ chiefly in how long they will observe pure viral persistence before inspecting the cervix, a choice reflecting local tolerance for delayed diagnosis, colposcopy capacity and confidence that women will return. The risk-based framework used in the United States sits at the more interventionist end of that range, referring at the second consecutive hrHPV-positive, cytology-negative result on the basis of an immediate CIN3+ risk just above 4.0%. The English and Australian pathways wait for a third consecutive positive result, and the Dutch programme may generate no colposcopy at all while cytology remains normal. The 2026 WHO genotyping guideline adds a further axis, not a further disagreement. It conditions the algorithm on programme follow-up and treatment capacity. In the strategy it prefers for adequately resourced settings, genotype allocates ablative treatment to eight of the twelve carcinogenic types and not merely diagnostic intensity [47]. Figure 2 sets out the modifiers that determine where an individual woman sits within these frameworks. It is an interpretive synthesis of the evidence reviewed here, not a guideline and not an algorithm for use in place of the recommendations in force in a given programme.

### 9.2. What Remains Conditional

Several questions central to daily practice remain unanswered by the literature identified here. No randomised comparison of surveillance intervals after a negative assessment was found, and the endpoint of surveillance, whether two consecutive negative HPV tests, a fixed number of years or an age threshold, is set by convention. We identified no study directly quantifying the incremental yield of repeating colposcopy after one adequate negative examination. The available observations are indirect, such as a series in which all high-grade lesions among women with an initial positive HPV test and no colposcopic or histological abnormality were diagnosed only after the second HPV test [99]. Whether risk estimates generated in prevalent-screen co-testing populations transfer to women with documented persistence and a prior negative assessment is assumed and not demonstrated. Nor did our searches return data describing the net effect, in women without confirmed CIN2+ specifically, of the ablative treatment that WHO guidance permits within screen-and-treat and within the genotype-directed algorithms it presents [11,47]. Finally, no trial of an agent directed at the infection has yet used CIN3+ or cancer as an endpoint, so whether short-term virological effects translate into clinical benefit is unknown.

### 9.3. Methodological Boundaries and Sources of Uncertainty

The evidence base is characterised by several recurring sources of heterogeneity and uncertainty. Definitions of persistence differ in ways that demonstrably alter effect estimates [14,15]. Most triage validation was performed at a single screening episode and not in persistently positive women, so predictive values are transported and not derived. Many estimates of colposcopic accuracy, particularly those using colposcopically directed biopsy as the reference standard, are vulnerable to verification bias, since the colposcopic impression determines the biopsy against which it is judged; the same consideration applies to the artificial-intelligence literature, which inherits that reference standard. The diagnostic-accuracy studies quoted here differ in reference standard, in biopsy protocol and in case mix, so their sensitivities are not strictly comparable and were not pooled. CIN3 remains the working surrogate endpoint despite evidence that it under-represents the precursors of HPV18- and HPV45-associated adenocarcinoma [26,41].

This review was designed as a structured narrative synthesis rather than a systematic review. Study selection was therefore question-driven, no quantitative pooling or formal certainty-grading framework was applied, and the searches were intended to identify the most clinically informative evidence rather than to provide exhaustive systematic retrieval. Statements regarding evidence gaps refer to the evidence identified through the described search process and should not be interpreted as proof of absence. The numerical ranges presented are descriptive summaries of the individual studies cited rather than pooled estimates. Programme-level outcome data, interval-cancer rates and cost-effectiveness analyses, all of which bear on whether any change in testing volume is justified, were outside its scope.

Within these methodological boundaries, the review provides an integrated clinical synthesis of natural-history risk, diagnostic performance and intervention evidence, domains that are frequently considered separately. It separates viral persistence from a lesion diagnosis, replaces the absolute notion of a lesion-free cervix with the more clinically precise concept of unconfirmed CIN2+, and keeps the three clinical states distinct throughout, so that each quantitative estimate is attached to the state that generated it. It compares the competing international management logics directly, including the 2026 revision of global guidance, and separates guidance written for individualised management from guidance written for programmes operating under capacity constraints. It asks explicitly whether the intensity of diagnostic assessment is proportionate to residual absolute risk. Finally, it evaluates the intervention literature not for internal validity alone but for transferability to the woman without confirmed CIN2+, which is the question a clinician facing her actually has to answer.

### 9.4. Research Priorities

Progress requires studies designed around this population and not borrowed from adjacent ones. A reporting standard specifying type-specific detection, a minimum 12-month interval and the assay used would make persistence estimates comparable across cohorts. Prospective cohorts should recruit at the second consecutive positive test with a documented negative assessment, follow women for CIN3+ and cancer over at least ten years, and bank specimens so that dual stain, extended genotyping and methylation can be evaluated in the same population against the same endpoints. Surveillance intervals of 12, 24 and 36 months should be compared in randomised fashion, but only within guideline-permitted risk strata in which genuine clinical equipoise exists; randomisation must never withhold colposcopy or treatment from a woman whose calculated risk exceeds an established action threshold. Adherence and patient-reported outcomes belong among the co-primary endpoints, since attrition is itself a determinant of programme effectiveness [93]. Methylation thresholds require prospective external validation across assays, populations and specimen types, including self-collected samples, fixed in advance [57]. Diagnostic-accuracy studies of computational colposcopy should be designed with an excisional or protocol-driven reference standard in cytology-negative women. Such designs would minimise the verification bias inherent in studies relying primarily on colposcopically directed biopsy as the reference standard. Products proposed for viral clearance should ideally undergo blinded, independently funded randomised evaluation before routine clinical adoption, and the field now needs trials powered for CIN3+ rather than for short-term loss of HPV detectability, with follow-up long enough to show whether an early virological difference persists. Diagnostic excision in the woman with an unassessable transformation zone is currently performed without evidence on its comparative yield, its effect on subsequent HPV status or its complication profile. It is an obvious candidate for randomised comparison against intensified molecular surveillance. Genotype risk tiers derived in unvaccinated cohorts require recalibration as vaccinated birth cohorts enter screening, and empirical, not modelled, criteria are needed for exiting screening while hrHPV remains detectable [69].

## 10. Conclusions

A woman with persistent high-risk HPV infection and no confirmed CIN2+ has no histologically confirmed high-grade treatment target. That is not the same as proof that she has no disease, and in state 1 the cervix has not been examined at all. Residual risk of occult or subsequently detected CIN3+ persists, and its magnitude depends on genotype, on the duration and type-specificity of persistence, on her previous screening history and on how complete the diagnostic assessment actually was. She is best understood as occupying a heterogeneous but quantifiable risk state and not as carrying a diagnosis. The probability of underlying or subsequent CIN3+ is determined principally by genotype and by how long the infection has persisted. The reassurance provided by a negative morphological assessment depends on examination completeness and on the known sensitivity of the diagnostic pathway: pooled colposcopic sensitivity for CIN2+ was approximately 75% in heterogeneous referral populations, and one selected retrospective series of cytology-negative hrHPV-positive women reported sensitivity near 50% [34]. Management is therefore better calibrated to that probability than to the presence or absence of a visible abnormality.

Four positions follow from that reading of the evidence. They are the authors’ own and are not offered in place of the recommendations in force in any programme. Management should be calibrated to the woman’s diagnostic state, to genotype-specific risk and to the action thresholds of the programme in force. Surveillance remains appropriate after an adequate negative assessment while residual risk stays below the applicable intervention threshold. HPV16 or HPV18 positivity, or repeated hrHPV-positive results with negative cytology, may trigger earlier colposcopic assessment depending on the programme, and a shortened interval is also appropriate for women living with HIV. Among the additional risk-stratification tools, extended genotyping [46,47] and p16/Ki-67 dual-stain cytology [51] currently have the strongest clinical role, being the only options carried through to guideline-adopted management rules. Methylation is promising and has long-term negative predictive data extending to 14 years, but it requires context-specific validation, and no programme-level guideline identified here has adopted it. These tools allocate diagnostic intensity more precisely; they do not substitute for programme-specific management. We identified no study measuring the incremental yield of a second colposcopy in a previously normal cervix. Treatment directed solely at persistent viral positivity is not currently supported. Controlled evidence exists but is sparse and conflicting: two randomised trials published in 2026 reached opposite short-term virological conclusions [83,84], the observational signal for thermal ablation plus a topical agent comes from a self-selected CIN1 population [85], and no trial has yet used CIN3+ or cancer as an endpoint. Short-term loss of HPV detectability is not equivalent to prevention of CIN3+. Excision here is a diagnostic procedure for a cervix that cannot be adequately assessed and not a therapy for infection. It carries a depth-dependent obstetric cost in women of reproductive age and a substantial residual rate of continuing HPV positivity in older women.

Persistence is not benign simply because it is not treatable. In two long-followed United Kingdom cohorts, risk continued to accrue for up to 30 years after a positive test [69]. Persistent hrHPV positivity should therefore be considered explicitly when programme-specific screening-exit criteria are applied, since the available cohort evidence does not support assuming that age alone normalises residual risk. Equally, the emergence of controlled intervention data is not by itself a reason to treat. Current evidence does not support routine treatment aimed solely at eradicating HPV in women who remain eligible for guideline-defined surveillance. The most clinically consequential remaining evidence gaps concern the optimal surveillance interval, the incremental yield of repeated diagnostic assessment, management of an inadequately assessable cervix, screening-exit criteria, and prospective validation of existing triage tools after documented persistence.

## Figures and Tables

**Figure 1:**
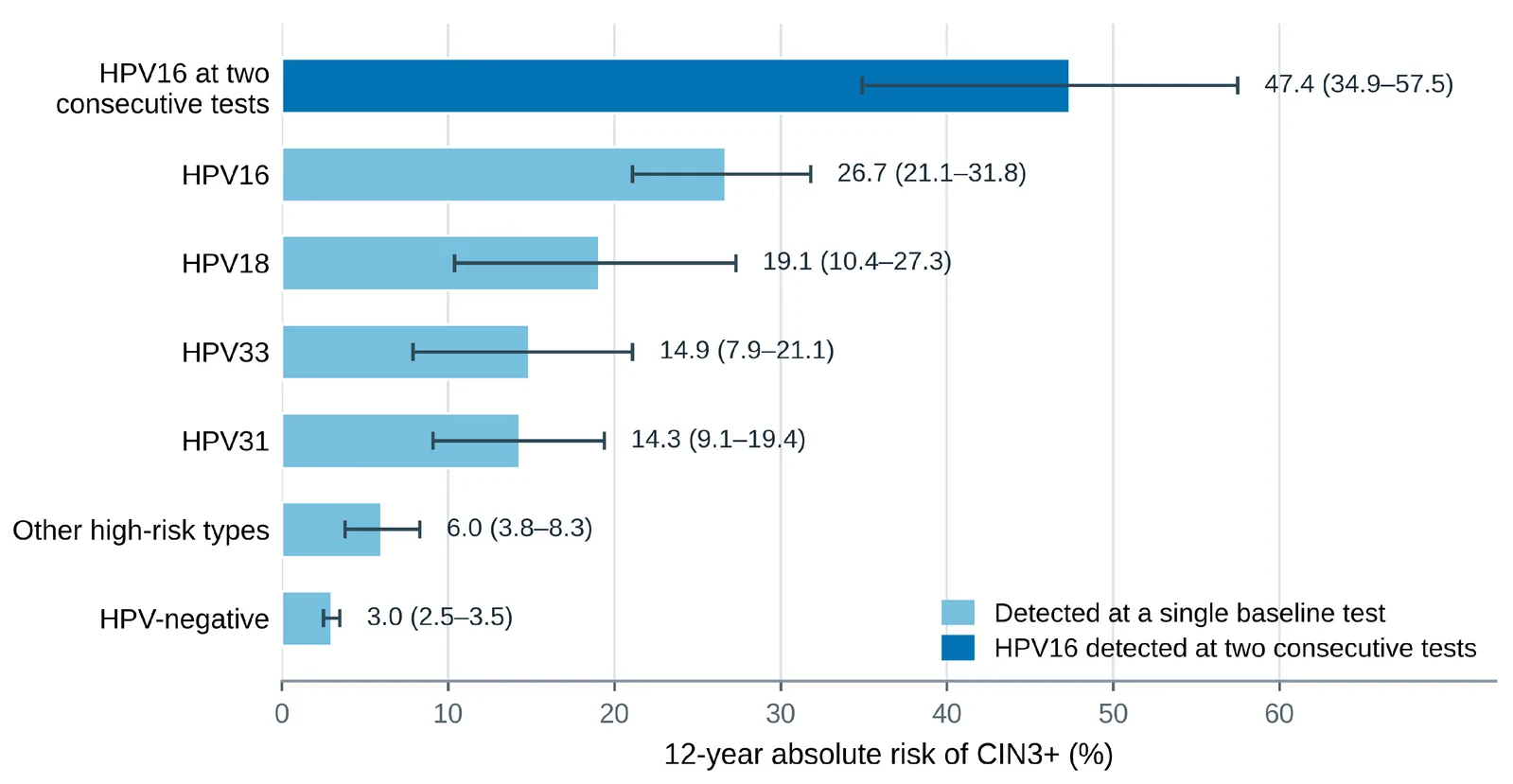
Illustrative long-term risk of cervical intraepithelial neoplasia grade 3 or worse (CIN3+) by HPV genotype and by documented HPV16 persistence. All estimates come from a single Danish population-based cohort of 8656 women followed for up to 13.4 years [4]. Because the endpoint, the time horizon and the source population are identical across bars, the values are internally comparable; they are not pooled estimates and should not be transported to other populations. Bars show the absolute 12-year risk and horizontal lines the 95% confidence interval, with the point estimate and interval printed beside each bar. Light bars denote a genotype detected at a single baseline test, together with the HPV-negative reference group, in which no genotype was detected. The dark bar denotes HPV16 detected at two consecutive examinations, and the gap between it and the corresponding light bar is the risk difference associated with documented HPV16 persistence in this cohort. Persistence is illustrated for HPV16 only: the figure does not demonstrate a genotype-by-persistence interaction across genotypes, because equivalent persistence estimates were not reported for the other types shown. The comparison is observational and no causal interpretation is implied. “Other high-risk types” comprises the carcinogenic genotypes other than HPV16, 18, 31 and 33. CIN3+, cervical intraepithelial neoplasia grade 3 or worse; HPV, human papillomavirus.

**Figure 2:**
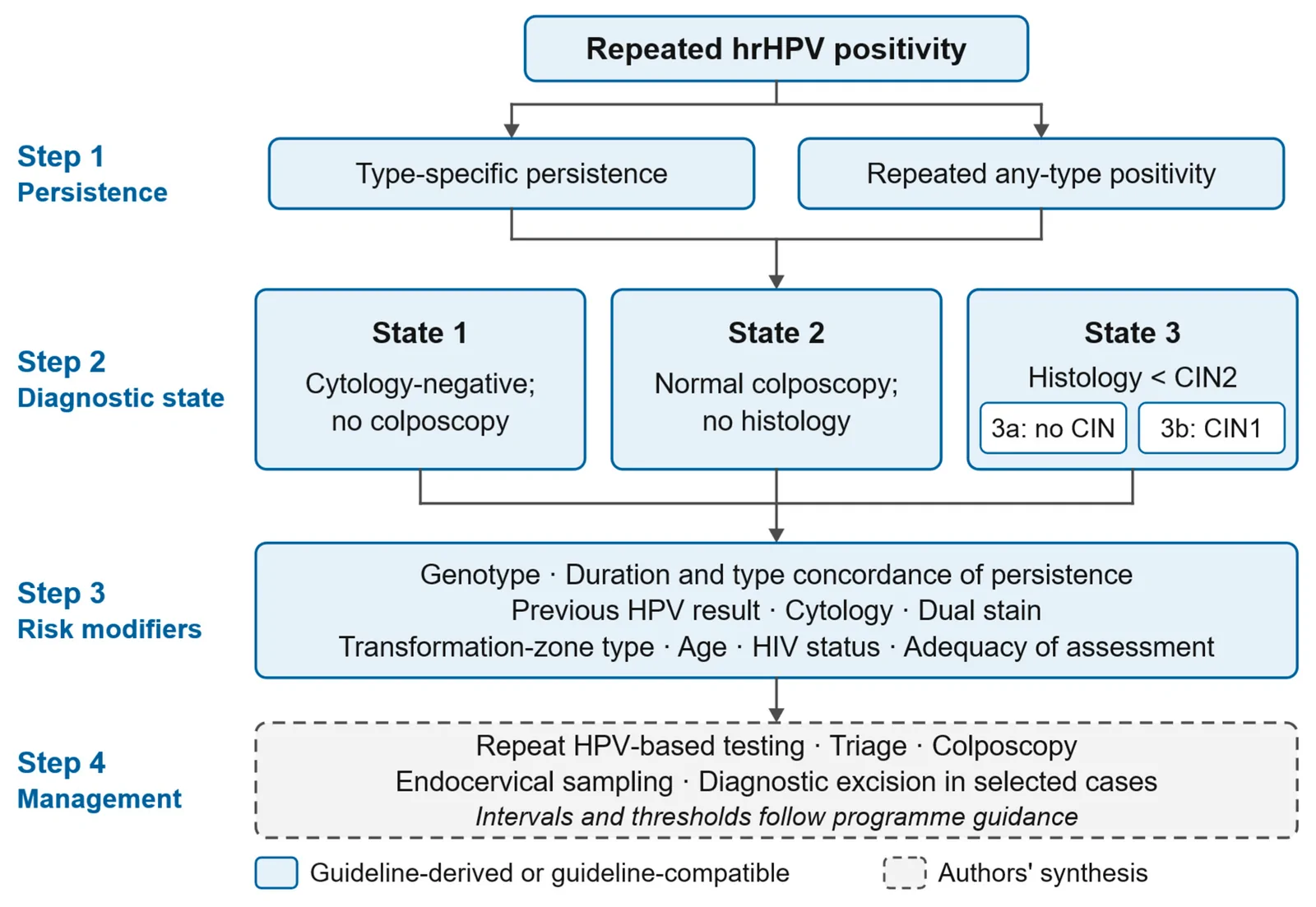
Evidence-informed framework for risk stratification and diagnostic management in persistent high-risk HPV (hrHPV) positivity. The framework organises the evidence reviewed here into four sequential considerations; it does not prescribe a management algorithm. Step 1 separates type-specific persistence, which can only be established where genotyping is available, from repeated any-type positivity, where it cannot; the two are not equivalent and carry different risk estimates (Section 2.1). Step 2 identifies which of the three diagnostic states defined in Section 2.2 the woman occupies, since absence of confirmed CIN2+ denotes materially different residual risks depending on whether the cervix has been inspected and sampled. State 3 is subdivided descriptively into 3a (negative or inflammatory histology) and 3b (histologically confirmed CIN1). Step 3 lists the modifiers that shift absolute CIN3+ probability within any of those states, including dual-stain cytology where validated and available. Step 4 sets out the management options available; the interval, the referral threshold and the treatment threshold are determined by the guidance in force in each programme and are deliberately not specified here. Triage may be molecular or cytological, and endocervical sampling applies where the squamocolumnar junction is not fully visible. Diagnostic excision is reserved for cases in which disease cannot otherwise be adequately excluded and is not a treatment for viral persistence. Outline style denotes provenance. Boxes with a solid outline contain elements whose content is drawn from, or is directly compatible with, published guideline recommendations [8,9,11,39,46,47,51,60,61]. The three-state taxonomy used in Step 2 and the sequencing of the four steps are the authors’ own organising devices and are defined in Section 2.1 and Section 2.2. The box with a dashed outline in Step 4 represents the authors’ interpretative synthesis of the evidence reviewed. Arrows denote the sequence of considerations and carry no causal or biological meaning. CIN, cervical intraepithelial neoplasia; CIN1, cervical intraepithelial neoplasia grade 1; CIN2, cervical intraepithelial neoplasia grade 2; CIN2+, cervical intraepithelial neoplasia grade 2 or worse; CIN3+, cervical intraepithelial neoplasia grade 3 or worse; HIV, human immunodeficiency virus; HPV, human papillomavirus; hrHPV, high-risk human papillomavirus.

**Table 1:** Triage options for the hrHPV-positive woman without confirmed CIN2+.

Triage Option	What It Measures	Population and Clinical State Evaluated	Reported Performance	Risk After a Negative Result (Endpoint, Horizon)	Evidence and Guideline Status	Principal Limitation in This Population
Repeat hrHPV test at 12 months [16,20]	Continued viral detection	Mixed screening populations, State 1	47% remain positive at 1 year (co-tested screening population); 61% at a median of 1.8 months, specificity < 50%	CIN3+, 5 years: 2.0% after a negative repeat test versus 0.2% in doubly negative women [31]	Guideline-adopted; the basis of every programme algorithm	Cannot distinguish persistence from reinfection or latency; poor specificity at short intervals
Extended genotyping [45,46,47,60,61]	Type-specific oncogenic potential	Screening populations, State 1; hrHPV-positive, cytology-negative subgroups	CIN3+ immediate/3 years: 8.4% and 11.5% for HPV16; 4.0% and 8.5% for HPV18; 1.8% and 2.9% for intermediate types; 0.4% and 0.9% for HPV56/59/66	CIN3+, 24 months: as low as 0.4% for the lowest-risk types with negative cytology, a group that included genotyping-assay-negative women (0.5% when they were excluded)	Guideline-adopted (United States enduring guidelines; WHO 2026 genotype groups); partial genotyping only in Australia and the Netherlands	Genotype distribution varies regionally and will shift as vaccinated cohorts enter screening; tiers derived in unvaccinated populations
Cytology [5,44]	Morphological abnormality	Persistent-hrHPV referral cohort (detection figures); co-tested screening population (risk figure)	HSIL confirmed in 42/156 women with ASC-US versus 46/337 with normal cytology at referral for persistent hrHPV	CIN3+, immediate: 2.1% for hrHPV-positive, cytology-negative results	Guideline-adopted; universal	Modest reproducibility for low-grade interpretations; insensitive for glandular disease
p16/Ki-67 dual stain [48,49,50,51,62]	Cell-cycle deregulation	hrHPV-positive screening populations, State 1	Sensitivity for CIN3+ 91.9% for HPV16/18 and 86.0% for other genotypes [50]; about one-third fewer colposcopies than cytology [49]	CIN2+ 3.6% (95% CI 2.9–4.4) at the index examination; CIN3+ 0.75% immediate and 1.5% at 3 years without genotyping	Guideline-adopted (United States); permitted WHO triage option since 2024, but not preferred under the 2026 WHO guideline [47]	Requires cytology infrastructure and trained readers; not validated on self-collected samples
Host and viral DNA methylation [53,54,55,56,57]	Epigenetic silencing accompanying transformation	Mixed triage and screening populations, State 1; one post hoc analysis of a randomised screening trial	Pooled sensitivity for CIN3+ 71.1% at fixed 70% specificity; 95% for cancer; discriminates future CIN3 from samples taken about 5 years before diagnosis	Cervical cancer, 14 years: cumulative incidence 1.7% after a negative test versus 2.4% after negative cytology (risk difference 0.71%, 95% CI 0.16–1.4)	Externally validated across cohorts; not adopted in the guidance reviewed here	Thresholds not standardised across assays; lower sensitivity for prevalent CIN2
E6/E7 messenger RNA [58]	Transcriptional activity of viral oncogenes	Routine screening population, State 1	No significant sensitivity difference from DNA testing, with significantly higher specificity and positive predictive value	Not established after documented persistence; CIN2+ at screening: an estimated 5.8% among Hybrid-Capture-2-positive, messenger RNA-negative women (verification-bias adjusted; single study)	Validated and commercially available as a primary screening assay; no triage rule after documented persistence in the guidance reviewed here	Not evaluated as a triage step after documented persistence
Viral integration status [59]	Physical state of the viral genome	Single Chinese screening cohort, State 1	Specificity for CIN3+ 94.5% versus 63.8% for cytology at equivalent sensitivity 70.5%	CIN3+, immediate: 2.2% among integration-negative women, who comprised 90.1% of the cohort	Single-cohort, hypothesis-generating	No external validation; no standardised assay

Clinical states are those defined in Section 2.2. With the exception of the cytology row, whose detection figures come from a persistent-hrHPV referral cohort, the estimates in this table were generated in hrHPV-positive women at a single screening episode and not in women with documented type-specific persistence following a previous negative or non-high-grade cervical assessment. They are transported into this population, not derived within it, and diagnostic performance measured in one population should not be assumed to transfer unchanged to another; predictive values in particular move with prevalence. Endpoints, time horizons and reference standards differ between rows and are stated in each cell, so the columns are not a like-for-like comparison and the options should not be ranked from them. ASC-US, atypical squamous cells of undetermined significance; CI, confidence interval; CIN2+/CIN3+, cervical intraepithelial neoplasia grade 2 or worse/grade 3 or worse; HPV, human papillomavirus; hrHPV, high-risk human papillomavirus; HSIL, high-grade squamous intraepithelial lesion; WHO, World Health Organization.

**Table 2:** Recommended management of high-risk human papillomavirus (hrHPV)-positive women without confirmed CIN2+: comparison of current guidance, by issuing body.

Guidance	hrHPV-Positive, Negative Cytology, First Occurrence	HPV16- or HPV18-Positive, Negative Cytology	Persistent hrHPV Positivity with Persistently Normal Cytology	After Colposcopy at Which CIN2+ Was Not Found	Excision or Ablation for Persistent hrHPV Without Histological CIN2+	Extended Genotyping or Dual-stain Triage
ASCCP, United States (2019 guidelines, with 2021 correction, 2024 consolidated update and 2025 Enduring Guidelines for extended genotyping)	Surveillance at 1 year; immediate CIN3+ risk 2.1%, below the 4.0% colposcopy threshold [8,44]	Colposcopy recommended; HPV18 an explicit exception to the risk threshold, on grounds of cancer risk [8]	Colposcopy. hrHPV-positive, cytology-negative results at two consecutive annual visits carry an immediate CIN3+ risk just above the 4.0% threshold [8,44]. A separate clarification covers cases in which colposcopy had been recommended and not performed, and cases in which an exact risk estimate is unavailable [8,63]	After low-grade referral results, 1-year HPV-based surveillance, not immediate repeat colposcopy; separate recommendations after high-grade referral cytology [8]	Not recommended; treatment thresholds are ≥25% (acceptable) and ≥60% (preferred) immediate CIN3+ risk; for grade 1 cervical intraepithelial neoplasia persisting ≥ 2 years, observation is preferred but excision or ablation is acceptable [8]	Both adopted through the enduring-guideline process; dual-stain-negative women return at 1 year (immediate CIN3+ risk 0.75% where genotyping is not available) unless HPV16/18-positive [46,51]
European Commission Initiative on Cervical Cancer (first recommendations, 2025)	Not addressed; the first recommendation set covers the primary screening test and the start and stop ages only [65]	Not addressed [65]	Final recommendations addressing triage, genotyping, surveillance and referral were not publicly available on the EC-CvC guideline portal by the closing date of this review	Not addressed	Not addressed	Not addressed
European quality-assurance guidance, 2015 supplements (legacy)	Repeat testing after ≥6–12 months; direct colposcopy explicitly not recommended [64]	No genotype-specific rule; management driven by triage cytology [64]	Second repeat after ≥12 months, colposcopy or return to routine screening if triage cytology remains normal. Colposcopy if triage cytology at repeat testing is ASC-US or worse [64]	Not separately specified [64]	Not addressed [64]	Predates both; silence is not a recommendation against [64]
Netherlands (referral scheme in force since July 2022)	Repeat cytology at 12 months [61]	Partial genotyping used; direct referral is triggered at ASC-US or worse for HPV16/18 versus HSIL or worse for other genotypes [61]	Return to screening if cytology remains normal at 12 months [61]	Not addressed; referral is cytology-driven [61]	Not addressed [61]	Partial genotyping only; extended genotyping and dual stain not used [61]
England (NHS cervical screening care pathway)	Repeat test at 12 months [9]	Genotyping not used in the routine pathway [9]	Colposcopy at the third consecutive hrHPV-positive result, irrespective of cytology [9]	Recall at 36 months if adequate colposcopy shows no cervical intraepithelial neoplasia, or at 12 months if grade 1; repeat colposcopy at 12 months if inadequate [9]	Not recommended, except that large loop excision of the transformation zone may be considered, by patient choice, after a repeat inadequate colposcopy [9]	Neither used [9]
Australia (National Cervical Screening Program)	Repeat HPV test at 12 months for genotypes other than HPV16/18 [60]	Colposcopy regardless of reflex cytology [60]	Colposcopy at 24 months if any oncogenic type persists; at 12 months in defined higher-risk groups [60]	HPV16/18 with negative cytology and normal colposcopy: 12-month follow-up, then, since 2025, optional further 12-month retest instead of rereferral [60]	Not recommended [60]	Partial genotyping only [60]
Canada (2023 colposcopy guideline, including risk-based entry criteria for colposcopy)	Not addressed for genotypes other than HPV16/18 [10]	Colposcopy regardless of cytology [10]	Not specifically addressed; annual colposcopic review until two consecutive negative HPV tests are obtained or histological HSIL is identified is specified for high-grade referral cytology [10]	Discharge to 12-month HPV-based screening, directly after low-grade referral cytology and, after high-grade referral cytology, once HPV is negative on two consecutive tests [10]	Not recommended; surveillance instead [10]	Neither specified [10]
World Health Organization (2021 second edition; 2024 dual-stain update; 2026 genotyping guideline)	Population-level strategy conditioned on programme capacity: either screen-and-treat or screen, triage and treat, both conditional recommendations [11]	Under the 2026 genotyping guideline, groups 1a (HPV16) and 1b (HPV18, HPV45) sit at the top of the genotype hierarchy; in the preferred algorithm for settings with adequate follow-up and treatment capacity, eligible women in groups 1a, 1b and 1c receive ablative treatment [47]	Retest at 24 months after a negative triage result; 12 months for women living with HIV [11]. The 2026 guideline does not address documented persistence after a previous negative assessment	For HPV-based screening, colposcopy is one of the triage tests: retest at 24 months after a negative triage result (12 months for women living with HIV). Retest at 12 months applies after cytology-based screening with normal colposcopy [11]	Permitted as a population-level, resource-stratified conditional recommendation, not as individualised management after a negative diagnostic assessment. Preceding morphology-based diagnostic triage and histological confirmation are omitted, but visual assessment of the cervix is still required to establish ablative eligibility and to exclude suspected cancer [11,47]	Extended or limited genotyping suggested over treating all or triaging all HPV-positive women in settings with ≥60% follow-up capacity. Among the extended-genotyping algorithms, treating groups 1a, 1b and 1c and triaging group 1d is preferred over more restrictive alternatives in which fewer groups are treated [47]. Dual stain permitted since 2024, retesting at 24 months after a negative result [62]; since 2026, visual inspection with acetic acid or colposcopic impression is suggested in preference to cytology or dual stain where an additional triage test is used [47]

Sources and versions are stated in the first column. The rows are not directly comparable in purpose. The ASCCP and European documents govern individualised management within organised screening. The Canadian guideline governs entry to and practice within the colposcopy pathway. WHO guidance sets population-level, resource-stratified strategies for national programmes, conditioned on follow-up and treatment capacity and not on an individual woman’s diagnostic history, apart from fixed retesting intervals. Programmes also differ in their use of persistence: England, Australia and the United States act on repeated any-type positivity, whereas the Netherlands acts on repeat cytology. Genotype-dependent rules apply only where genotyping is performed; England does not genotype in the routine pathway, and the Netherlands and Australia use partial genotyping only. ASC-US, atypical squamous cells of undetermined significance; ASCCP, American Society for Colposcopy and Cervical Pathology; CIN2+/CIN3+, cervical intraepithelial neoplasia grade 2 or worse/grade 3 or worse; EC-CvC, European Commission Initiative on Cervical Cancer; HIV, human immunodeficiency virus; HPV, human papillomavirus; hrHPV, high-risk human papillomavirus; HSIL, high-grade squamous intraepithelial lesion; NHS, National Health Service; WHO, World Health Organization.

## Data Availability

No new data were created in this study. All data discussed are contained within the cited publications and in the Supplementary Materials. The literature sources and principal search concepts are summarised in Table S4.

## Supplementary Materials

The following supporting information can be downloaded at: https://www.mdpi.com/article/10.3390/diagnostics16213482/s1. Table S1: Longitudinal evidence linking high-risk HPV detection and persistence to cervical precancer and cancer risk; Table S2: Detection yield of colposcopic and histological assessment in high-risk HPV-positive women, with the population spectrum and principal limitation of each source; Table S3: Interventions evaluated for eradication of high-risk HPV infection or prevention of subsequent lesions and their applicability to the woman without confirmed CIN2+; Table S4: Literature sources and search concepts used in this narrative review; Table S5: SANRA item mapping. Reference numbering in the supplementary file follows the main manuscript.

## Abbreviations

The following abbreviations are used in this manuscript:

Abbreviation	Definition
AIS	adenocarcinoma in situ
ASC-H	atypical squamous cells, cannot exclude high-grade squamous intraepithelial lesion
ASC-US	atypical squamous cells of undetermined significance
ASCCP	American Society for Colposcopy and Cervical Pathology
CI	confidence interval
CIN	cervical intraepithelial neoplasia
CIN2+	cervical intraepithelial neoplasia grade 2 or worse
CIN3+	cervical intraepithelial neoplasia grade 3 or worse
HIV	human immunodeficiency virus
HPV	human papillomavirus
hrHPV	high-risk human papillomavirus
HSIL	high-grade squamous intraepithelial lesion
LSIL	low-grade squamous intraepithelial lesion
NHS	National Health Service
NILM	negative for intraepithelial lesion or malignancy
WHO	World Health Organization
