# Supplementary material for: Persistent High-Risk HPV Positivity Without Confirmed CIN2+: Diagnostic Uncertainty, Risk Stratification, and Management—A Narrative Review
Source: Diagnostics (Basel). 2026 Oct 24;16(21):3482. doi: 10.3390/diagnostics16213482 (PMC13762138; doi:10.3390/diagnostics16213482)
Supplement: Supplementary file 1 [file diagnostics-16-03482-s001.zip › diagnostics-4596353-supplementary.pdf]

Supplementary Materials

Persistent High-Risk HPV Positivity without Confirmed CIN2+: Diagnostic Uncertainty, Risk Stratification, and Management—A Narrative Review

Reference numbers correspond to the reference list of the main manuscript. Only the references cited in these tables are reproduced below. Clinical states 1, 2 and 3, with the descriptive subcategories 3a and 3b, are those defined in Section 2.2 of the main manuscript. Abbreviations are as defined in the main manuscript; in addition, AGC, atypical glandular cells; ChiCTR, Chinese Clinical Trial Registry; IgY, immunoglobulin Y; mITT, modified intention-to-treat; PCR, polymerase chain reaction; RIVM, National Institute for Public Health and the Environment; ROB-2, version 2 of the Cochrane risk-of-bias tool for randomised trials; ROBINS-I, Risk Of Bias In Non-randomised Studies of Interventions; RR, relative risk; SANRA, Scale for the Assessment of Narrative Review Articles.

Table S1. Longitudinal evidence linking high-risk HPV detection and persistence to cervical precancer and cancer risk.

Study, design and setting	Population	Follow-up	Principal absolute risk estimates	Persistence definition, clinical state and measure type
Prospective population-based cohort, Denmark [4]	8656 women	Up to 13.4 years	12-year CIN3+ risk: HPV16 26.7% (95% CI 21.1–31.8); HPV18 19.1% (10.4–27.3); HPV33 14.9% (7.9–21.1); HPV31 14.3% (9.1–19.4); other high-risk types 6.0% (3.8–8.3); HPV-negative 3.0% (2.5–3.5). HPV16 detected at two consecutive examinations: 47.4% (34.9–57.5)	Type-specific detection at two consecutive examinations; State 1; cumulative absolute risk
Prospective community-based cohort, Taiwan [25]	10,123 women with normal cytology at entry; 68 incident cancers	16 years	Cumulative cervical cancer risk: HPV16 13.5%; HPV58 10.3%; other carcinogenic types 4.0%; HPV-negative 0.26%. Persistent carcinogenic infection versus persistently negative: hazard ratio 75.4 (95% CI 31.8–178.9)	Type-specific persistence across serial visits; State 1; cumulative absolute risk and hazard ratio
Longitudinal cohort nested in an organised co-testing programme, United States [22]	11,573 hrHPV-positive women aged 30–65; 14,158 type-specific infections	7 years	7-year CIN3+ risk: HPV16 22%; HPV18 and 45 >5%; HPV31, 33, 35, 52, 58 >5%; HPV39, 51, 56, 59, 68 <5%. Approximately 80% of infections undetectable within 3 years	Type-specific infections in hrHPV-positive women; State 1; cumulative absolute risk by genotype tier
Prospective population-based cohort, Costa Rica [27]	2282 sexually active women, two visits about 1 year apart	3–5 years	3-year cumulative CIN2+: positive–positive 17.0% (95% CI 12.1–22.0); negative–positive 3.4% (0.1–6.8); positive–negative 1.2% (−0.2 to 2.5); negative–negative 0.5% (0.1–0.9). Short-term HPV16 persistence: 5-year cumulative CIN2+ 40.8% (26.4–55.1)	Any-type positivity at two visits about 1 year apart; State 1; cumulative incidence
Prospective population-based cohort, prevalent versus incident infection, Costa Rica [23]	9175 women screened	7 years	66 of 85 cases of CIN2 or worse detected during follow-up arose from infections present at enrolment; newly detected infections carried very low absolute risk regardless of age	Prevalent versus incident detection; State 1; cumulative absolute risk
Post-hoc analysis of the control arm of a randomised vaccine trial, multinational [3]	4825 women; 16,785 infections	48 months	Cumulative clearance 53% (12 months), 79% (24), 87% (36), 89% (48). Progression of 6-month persistent infection to CIN2+, hazard ratio versus non-oncogenic types: HPV16 10.44 (95% CI 6.96–15.65); HPV33 9.65 (5.97–15.60); HPV31 5.68 (3.50–9.21); HPV45 5.38 (2.87–10.06); HPV18 3.87 (2.38–6.30)	All incident and prevalent infections, not restricted to persistence; State 1; clearance proportions and progression hazard ratios
Cohort analysis nested in a randomised screening trial, Netherlands [31]	199 hrHPV-positive, cytology-negative women with a negative repeat test; 18,562 doubly negative controls	5 years	5-year CIN3+ risk 2.0% (4/199) versus 0.2% (41/18,562); p < 0.001	Any-type positivity with a negative repeat test; State 1; absolute risk after a negative result
Two prospective screening cohorts with registry linkage, United Kingdom [70]	47,625 and 24,496 women	30 and 17 years	15-year cumulative CIN3 risk after hrHPV positivity 11.7% and 12.0%; HPV16 18.2% and 23.9%. Invasive cancer risk rose for 30 years, reaching 2.5% (95% CI 1.3–4.5)	Single baseline positive test, not persistence; State 1; long-term cumulative risk
Prospective population-based cohort restricted to women with a histologically normal cervix, China [28]	1232 women with abnormal cytology; 357 (29.0%) hrHPV-positive at entry	2 years	Progression to CIN1+ 2.6% (32/1232); CIN2/3 0.8% (10/1232). Same genotype at follow-up: adjusted odds ratio 22.26 (95% CI 8.98–55.17); different genotype 3.92 (0.81–18.99); both versus persistently hrHPV-negative women, for progression to CIN1+	Same-genotype versus different-genotype detection at follow-up; State 3a (histological component only; hrHPV persistence not documented at entry); adjusted odds ratio
Population-based screening cohort with extended genotyping, Finland [21]	35,084 genotyped women; 2006 hrHPV-positive	0.5–4 years	884/2006 (44.1%) met a type-specific persistence definition; 197 (22.3%) of these women progressed to precancer. Persistence highest for HPV52 (52.2%), HPV58 (50.5%), HPV31 (50.4%), HPV16 (48.9%)	Type-specific positivity at two rounds; State 1 (negative or borderline baseline cytology); proportion persisting and proportion progressing
Prospective cohort nested in an organised screening programme, Italy [29]	1540 baseline hrHPV-positive women without CIN2+, retested at a median	To 24 months	All 11 CIN3 cases reported overall occurred in persistent infections, none in exclusively new infections; 3 CIN3 cases also reported among women positive on the screening HPV test but Onclarity-negative at retesting; published overall	Type-specific versus newly acquired infection at retesting; mixed diagnostic histories (negative cytology triage, or colposcopy without CIN2+), adjusted new-infection proportions reported

Study, design and setting	Population	Follow-up	Principal absolute risk estimates	Persistence definition, clinical state and measure type
	of 13.5 months		and subgroup CIN3 counts do not fully reconcile; 68/613 (11.1%) of women positive at retesting with available genotyping results were classified as having exclusively new infections	separately (11.5% and 15.1%, respectively), distinct from the crude 68/613; reclassification proportions

The estimates in this table are not all of the same kind. Cumulative absolute risks, cumulative incidences, hazard ratios, adjusted odds ratios and clearance proportions appear in different rows and are not interconvertible; the measure type is stated for each study. Persistence definitions also differ, and several rows report risk after a single detection and not after documented persistence. No single cohort was assembled across the full three-state phenotype defined in this review, although some rows represent an individual state more closely than others. Several estimates therefore require transport across differences in persistence definition, in diagnostic state and in whether any prior cervical assessment had been performed.

Table S2. Detection yield of colposcopic and histological assessment in high-risk HPV-positive women, with the population spectrum and principal limitation of each source.

Study, design and setting	Population	n	Yield	Comment	Population spectrum and principal limitation
Retrospective cohort within an organised programme, Finland [5]	Referred for persistent hrHPV with normal or borderline cytology	493	HSIL in 17.8% overall; 46/337 with normal cytology and 42/156 with ASC-US; 78/345 aged <50 years versus 10/148 aged ≥50	Most high-grade lesions lacked classical colposcopic features	Programme referral population enriched for persistence; verification bias, since biopsy followed colposcopic impression
Retrospective referral cohort, Germany [6]	hrHPV persistence at 1 year, or low-grade cytology with hrHPV	334 (237 with persistence)	CIN2+ 7.6% with normal cytology; 18% with ASC-US	Single colposcopy with directed biopsy or, for a type 3 transformation zone, endocervical curettage	Colposcopy referral population; retrospective, single centre; verification bias
Multicentre retrospective series, Germany [7]	Women aged ≥65 with persistent hrHPV and negative cytology	211	CIN2+ 20/182 (11.0%; reported as 10.6%) among women with a biopsy or excision specimen; half of CIN2+ cases HPV16-positive	Type 3 transformation zone in 185/211 (87.7%); sampling not possible in 35/211 (16.6%), so the analysed denominator is smaller than the enrolled cohort	Highly selected population aged ≥65 with a type 3 transformation zone in 87.7%; sampling impossible in 16.6%
Retrospective referral cohort, China [34]	Cytology-negative, hrHPV-positive women biopsied at colposcopy	372	CIN2/3 9.95%; invasive carcinoma 2.42%	Colposcopic sensitivity 50.0%, specificity 96.9%; a referral population, and the cancer rate is not generalisable to organised screening	Referral population with 2.42% invasive carcinoma; not a screening population; verification bias
Retrospective cohort, Türkiye [95]	Non-16/18 hrHPV, normal cytology	454 screened; 142 biopsied immediately and 312 with normal colposcopy followed, of whom 276 had 12-month follow-up data available; women without follow-up data were excluded from the follow-up analysis	Immediate biopsy: CIN2+ 7.0% (95% CI 2.9–11.1); deferred group: no CIN2+, of whom 32.2% remained hrHPV-positive	Allocation not randomised; with zero events the one-sided 95% upper bound in the deferred group is about 1.1%	Screening population, but biopsy restricted to women with an abnormal colposcopic impression, so the 7.0% denominator is that subgroup and not the whole cohort
Retrospective series of diagnostic excisions, Germany [87]	Excision for HPV persistence, repeated cytological abnormality, type 3 transformation zone or stenosis	89	Histological abnormality 53.9%; CIN2+ 25.8%; CIN3 10.1%; early invasive carcinoma 2.3%	Highly selected; excision performed because adequate sampling was impossible	Highly selected diagnostic-excision cohort; yields are not prevalence estimates for persistent hrHPV generally
Secondary analysis of a prospective biopsy study, United States [38]	Women undergoing colposcopy for abnormal cytology	181 with curettage	Endocervical CIN2+ 14.4% (95% CI 10.0–20.2); incremental yield beyond up to four directed biopsies 3.9% (1.9–7.8)	Incremental yield below 5% when colposcopy was normal or satisfactory	Referral population with abnormal cytology; incremental yield of curettage measured against directed biopsy
Retrospective paired-sampling study, Romania [40]	Type 3 transformation zone, paired ectocervical biopsy and curettage	690	Curettage-only CIN2+ 2.8% (19/690); HPV16/18 4.5% versus 1.3% for other types (p = 0.017)	22.1 curettages per additional CIN2+ with HPV16/18 versus 76.0 with other types	Referral population with 16.8% negative cytology and 48.0% CIN2+ prevalence; absolute yields not transportable; the genotype gradient is transferable
Retrospective diagnostic-accuracy study, China [35]	Women with type 3 transformation zone	764	Sensitivity 51.2%, specificity 96.5% for CIN2+	Quantifies the assessability problem in older women	Referral population restricted to type 3 transformation zone; reference standard

Study, design and setting	Population	n	Yield	Comment	Population spectrum and principal limitation
					obtained from a compartment that cannot be inspected
Prospective observational biopsy study, United States [33]	Women referred after abnormal screening	690	Sensitivity for HSIL 60.6% (one biopsy), 85.6% (two), 95.6% (three); 2–4% of HSIL found by non-targeted biopsy	Referral population; establishes the multiple-biopsy standard	Referral population; the benchmark for biopsy-number effects, not a population without confirmed CIN2+
Prospective diagnostic-accuracy study, United Kingdom [52]	Women scheduled for excision with hrHPV and type 3 transformation zone	101	Dual-stained cytology positive predictive value 76.1% against CIN2+ prevalence 35.9% (low-grade referral); dual-stained curettings 96.5% against 75.6% (high-grade referral)	Small; no CIN2+ after a negative dual stain in the low-grade referral group	Small, highly selected excision population
Retrospective cross-sectional study, Portugal [100]	Positive HPV test followed by repeat testing, minor or absent cytological abnormality, no colposcopic lesion and no dysplasia	141	6 women subsequently diagnosed with HSIL, all after the second HPV test	Small and retrospective; hypothesis-generating	Retrospective, small; hypothesis-generating only

Many estimates of colposcopic accuracy in this table, particularly those using colposcopically directed biopsy as the reference standard, are vulnerable to verification bias, since the colposcopic impression determines the biopsy against which it is judged. Rows drawn from diagnostic-excision cohorts and from referral populations with high lesion prevalence are identified as such; their CIN2+ and cancer yields are conditional on selection and must not be read as prevalence estimates for the general persistent-hrHPV population. Only the Finnish [5] and German [6,7] rows, and not the Turkish [95] or Portuguese [100] rows, were generated in women with documented persistence or repeated positivity and negative or borderline cytology; the Turkish and Portuguese cohorts were assembled at a single hrHPV-positive result, with persistence assessed only during follow-up, and the estimates in the remaining rows are transported from adjacent populations. Even the three derived rows are referral series, so their absolute yields remain conditional on selection.

Table S3. Interventions evaluated for eradication of high-risk HPV infection or prevention of subsequent lesions, and their applicability to the woman without confirmed CIN2+.

Intervention	Design	Population studied	Principal result	Applicability to persistent hrHPV without confirmed CIN2+	Comparator, blinding and primary endpoint
Bivalent HPV-16/18 vaccine [71]	Randomised controlled trial	2189 HPV DNA-positive women aged 18–25	Vaccine efficacy for clearance 2.5% (95% CI −9.8 to 13.5) at 6 months; −2.0% (−24.3 to 16.3) at 12 months	A randomised test of whether prophylactic HPV vaccination has a therapeutic effect on an already established infection; the result was null, with wide confidence intervals, in a younger population	Control vaccine; blinded; virological (clearance of HPV-16/18 at 6 and 12 months)
Bivalent vaccine, extended analysis [72]	Analysis of a randomised trial	1711 women with carcinogenic HPV at vaccination; 311 treated by excision	No increase in clearance of infections present at vaccination; no significant reduction in lesions arising from them	Indicates that any benefit is prophylactic and not therapeutic	Control vaccine; blinded; virological and lesion endpoints in infections present at vaccination
Adjuvant vaccination after excision [73]	Systematic review and meta-analysis (17 studies, 4 randomised)	33,181 women treated for CIN	Pooled relative risk of recurrent CIN2+ 0.38 (95% CI 0.29–0.51); I^2 = 58.6%; attenuated effect in randomised trials (meta-regression relative risk approximately 0.55)	Different population; estimate dominated by non-randomised cohorts; not transferable	Mostly non-randomised comparators; blinding absent in the cohorts; histological (recurrent CIN2+)
VGX-3100 therapeutic DNA vaccine [74]	Randomised, double-blind, placebo-controlled phase 2b trial	Biopsy-confirmed CIN2/3	Per-protocol regression 49.5% versus 30.6%; difference 19.0 percentage points (95% CI 1.4–36.6); p = 0.034	Target is a lesion, not an infection; no published phase 3 confirmation	Placebo; double-blind; histological (regression of CIN2/3)
GTL001 therapeutic vaccine [77]	Phase I safety and immunogenicity study	47 HPV16/18-positive women with normal cytology	Acceptable safety and immunogenicity; exploratory virological comparisons only	We identified one therapeutic vaccine study in this population; it was not an efficacy trial	Placebo-vaccine arm in a randomised double-blind part, plus an open-label part; safety and immunogenicity; virological comparisons exploratory
Topical imiquimod [75]	Randomised controlled trial	90 women with cervical HSIL	Regression to CIN1 or less 61% versus 23% (per protocol, 78 of 90 enrolled); p = 0.001	Studied in HSIL only	Excision without preceding treatment; unblinded; histological (regression of HSIL)
Topical 5-fluorouracil [76]	Randomised controlled trial	60 women with CIN2	Regression 93% versus 56% among women with a 6-month biopsy; intention-to-treat relative risk 1.62 (95% CI 1.10–2.56)	Studied in CIN2 only	Observation; unblinded; histological (regression of CIN2)
Oral green tea catechins (Polyphenon E) [78]	Randomised, double-blind, placebo-controlled phase II trial	98 women with persistent hrHPV and CIN1	Complete response 17.1% versus 14.6%; no effect on viral clearance	Closest study population (state 3b only); null	Placebo; double-blind; histological response and viral clearance

Intervention	Design	Population studied	Principal result	Applicability to persistent hrHPV without confirmed CIN2+	Comparator, blinding and primary endpoint
Oral probiotics [79]	Randomised, placebo-controlled trial	121 hrHPV-positive women selected by negative cytology, without histological verification	Clearance 58.1% versus 54.2%; log-rank p = 0.741	Null for the relevant endpoint	Placebo; blinded; virological (hrHPV clearance)
Carrageenan-based vaginal gel [80,81]	Randomised, double-blind, placebo-controlled phase IIB trial [80] and viral-load substudy [81]	Sexually active young women, not selected by cervical histology	Reduced acquisition of new infection without significantly faster clearance of established infection [80]; no significant reduction in HPV42 or HPV51 viral load in a substudy [81]	Prevents acquisition; no demonstrated clearance of existing infection	Placebo; double-blind; virological (incident infection primary, clearance secondary [80]; viral load in a substudy [81])
Coriolus versicolor-based vaginal gel [82,83]	Open-label randomised trial versus watchful waiting; remaining literature uncontrolled or retrospective	91 women with HPV-dependent cervical lesions (low-grade cytology with concordant colposcopic findings; no biopsies); 70 hrHPV-positive, 21 with low-risk HPV only	Primary composite of normal cytology with concordant colposcopy 84.9% versus 64.5% (p = 0.031); hrHPV clearance (secondary) 62.5% versus 40.0% in the pooled comparison (p = 0.076), significant for one of two dosing schedules	Pooled virological comparison not significant; colposcopy assessed unblinded; no histological endpoint	Watchful waiting; open-label; composite of cytology and colposcopy as primary endpoint, HPV clearance secondary; funded by the manufacturer
Alternatives to surveillance, overall [12]	Systematic review and meta-analysis	Women with persistent HPV after a positive screen	12 studies (4 cohorts, 8 randomised trials; 2135 records screened): 6 oral agents, 2 topical agents, 1 vaccine, 3 non-surgical device treatments. Some interventions, including vaginal gels, photodynamic therapy and certain oral agents, may bring forward hrHPV resolution and low-grade CIN regression versus natural clearance	Eligible population heterogeneous, spanning persistent hrHPV and low-grade disease up to CIN1 with variable diagnostic histories, so the pooled signal is not specific to any one of the three clinical states	Mixed comparators; blinding variable; mixed virological and histological endpoints; risk of bias assessed with ROBINS-I and ROB-2
PHPV® fusion-factor vaginal gel [84]	Randomised controlled trial, three arms (gel vs interferon vs no treatment), open-label, single centre	120 randomised / 110 mITT; persistent same-type hrHPV, high-grade lesions excluded, colposcopic biopsy before enrolment; negative/inflammatory histology in 50 (41.7%; state 3a) and CIN1 in 70 (58.3%; state 3b)	6-month hrHPV clearance 66.7% (gel) vs 45.9% (interferon) vs 20.6% (control); adjusted RR gel vs control 2.81 (95% CI 1.37–5.79; p = 0.005); interferon vs control 2.08 (0.98–4.44; p = 0.058), not significant; no serious adverse events	A histologically assessed population without confirmed CIN2+, corresponding to state 3 (states 3a and 3b); the accessible published results do not provide clearance estimates stratified by baseline histology, so differential efficacy between 3a and 3b cannot be established; endpoint short-term virological only, with no CIN3+ or cancer outcome and no data beyond 6 months	No treatment control; open-label; virological primary endpoint at 6 months; product supplied by the manufacturer; single centre; ChiCTR2200063608
Silicon dioxide-based vaginal gel [85]	Randomised controlled trial, parallel group, open-label, single centre	242 women, PCR-confirmed hrHPV, no indication for immediate treatment; baseline colposcopy with biopsy directed by colposcopic and clinical findings; CIN1/LSIL (state 3b) in 5, CIN2/HSIL in 1; high-grade referral cytology (ASC-H, AGC favouring neoplasia or HSIL) in 29 (12.0%); histology not reported for the remaining participants	Absence of detectable hrHPV DNA at 3 months 18% (gel) vs 21% (observation); no significant difference; patient-reported adverse events 32% vs 2%; lower satisfaction with gel	Highly relevant negative randomised evidence, although the enrolled population was not entirely restricted to the target phenotype because 29 participants (12.0%) had high-grade referral cytology and one had baseline CIN2/HSIL; did not reproduce the large short-term virological benefit reported in earlier product-related studies; results not reported by baseline histology, so no estimate is specific to state 3a or 3b	Observation control; open-label; virological primary endpoint at 3 months; investigators declared no conflicts of interest
Thermal ablation plus HPV-specific IgY vaginal gel [86]	Prospective observational cohort, non-randomised, self-selected treatment, single centre	237 women with persistent HPV for at least 1 year and histologically confirmed CIN1 (106 treated, 131 watchful waiting)	12-month lesion regression 65.1% vs 35.9% and HPV clearance 67.9% vs 35.1% (both $p < 0.001$); progression to CIN2+ 4.7% vs 8.4% (p = 0.267), not significant	Within the broad phenotype of persistent HPV without confirmed CIN2+, but representing state 3b only, not state 3a or women with no visible lesion; allocation self-selected, so confounding by indication cannot be excluded	Watchful-waiting comparator; unblinded; primary objectives of composite regression (HPV negativity with normal cytology, or non-CIN histology) and HPV clearance; incremental effect of the gel over ablation alone not separable

The population column distinguishes negative/inflammatory histology, CIN1, CIN2/3 and post-treatment populations, and separates randomised from non-randomised designs. Efficacy demonstrated in histologically confirmed CIN2/3 cannot be extrapolated to treatment of isolated persistent HPV positivity without confirmed high-grade disease. Where a product is commercially marketed for viral clearance, the comparator, the blinding, the funding or supply arrangement and the primary endpoint are stated so that the strength of the design is visible. Virological endpoints (loss of HPV detectability) are distinguished from histological endpoints (lesion regression); no trial in this table used CIN3+ or cervical cancer as an endpoint.

Table S4. Literature sources and search concepts used in this narrative review.

Resource	Coverage and interface	Principal topics covered
PubMed/MEDLINE	pubmed.ncbi.nlm.nih.gov	HPV persistence, cervical diagnostic assessment, biomarkers and CIN2+/CIN3+ risk
Europe PMC	europepmc.org	The same topics, extending to preprints, guideline annexes and records not yet indexed in MEDLINE
Scopus	titles, abstracts and keywords	The same topics, with emphasis on diagnostic-accuracy studies and non-MEDLINE-indexed European programme literature
Guideline and programme sources	Current versions	Hand searching of the issuing bodies compared in Section 6: ASCCP, NHS England, RIVM, Cancer Council Australia and the Australian Department of Health, the Gynecologic Oncology Society of Canada, the World Health Organization, the European Commission Initiative on Cervical Cancer and the European Commission quality-assurance documents
Reference-list searching	Retrieved guidelines and recent reviews	Reference lists of the guidelines retrieved above and of recent reviews on HPV persistence, triage and colposcopic accuracy
PubMed/MEDLINE and Scopus (intervention literature)	pubmed.ncbi.nlm.nih.gov and Scopus (titles, abstracts and keywords)	Interventions evaluated for persistent HPV infection and its clearance, including oral, topical, immunological and procedural approaches

The review is narrative and non-exhaustive, and no record counts or screening flow are reported. Sources were selected for relevance to the three questions stated in Section 1, rather than through a systematic eligibility and screening process.

Table S5. SANRA item mapping.

SANRA item	Criterion	How the criterion is addressed in this review
1. Justification of the article's importance	The importance of the topic for the readership is explained	Section 1 quantifies the population generated by primary HPV screening, the proportion in whom high-grade disease is not confirmed, and the divergence between programmes in managing them
2. Statement of concrete aims or questions	Aims or questions are stated concretely	Section 1 states three explicit questions and defines what falls outside the scope of the review
3. Description of the literature search	The search is described so that a reader can judge its adequacy	Section 1.1 describes the literature sources, broad thematic scope and principles guiding source selection. Table S4 summarises the principal sources and topics covered. The review is narrative and non-exhaustive.
4. Referencing	Statements are referenced to appropriate sources	Primary studies are cited for individual-study estimates and systematic reviews or meta-analyses for pooled estimates; guideline statements are referenced to the issuing sources, with version dates specified where available.
5. Scientific reasoning	Appropriate evidence is presented and alternative explanations discussed	Section 4 sets out four competing explanations for the same clinical picture; Sections 5 and 9 distinguish detection, risk stratification, prognostic enrichment and clinical utility, and identify where estimates are transported and not derived
6. Appropriate presentation of data	Relevant endpoint data are presented with appropriate measures of uncertainty	Absolute risks, denominators, time horizons and 95% confidence intervals are reported where available in the cited sources; endpoints and composite outcomes are distinguished.

SANRA is a quality-appraisal instrument for narrative reviews. This table records where each criterion is addressed in the manuscript; it does not imply that systematic-review methodology was used, and no score is assigned.

References Cited in the Supplementary Materials

3. Jaisamrarn, U.; Castellsagué, X.; Garland, S.M.; Naud, P.; Palmroth, J.; Del Rosario-Raymundo, M.R.; et al. Natural history of progression of HPV infection to cervical lesion or clearance: Analysis of the control arm of the large, randomised PATRICIA study. PLoS ONE 2013, 8, e79260. https://doi.org/10.1371/journal.pone.0079260

4. Kjær, S.K.; Frederiksen, K.; Munk, C.; Iftner, T. Long-term absolute risk of cervical intraepithelial neoplasia grade 3 or worse following human papillomavirus infection: Role of persistence. J. Natl. Cancer Inst. 2010, 102, 1478–1488. https://doi.org/10.1093/jnci/djq356

5. Hulmi, J.; Virtanen, A.; Sadeluoto, H.; Tarkkanen, J.; Kalliala, I.; Heinonen, A. Histological outcomes and colposcopic findings in patients with persistent high-risk human papillomavirus infection without major cytological findings. Am. J. Obstet. Gynecol. 2026, 235, 356–364. https://doi.org/10.1016/j.ajog.2026.03.016

6. Wittenborn, J.; Kupec, T.; Iborra, S.; Najjari, L.; Kennes, L.N.; Stickeler, E. CIN2+ detection in high-risk HPV patients with no or minor cervical cytologic abnormalities: A clinical approach validated by machine learning. Arch. Gynecol. Obstet. 2023, 307, 881–890. https://doi.org/10.1007/s00404-023-06953-6

7. Kuenkel, E.; Jaeger, A.; Bohlmann, I.; Bergauer, F.; Kuehler-Obbarius, C.; Prieske, K.; et al. The use of colposcopy for triage in HPV-positive women aged 65 years and older. Arch. Gynecol. Obstet. 2024, 309, 1561–1567. https://doi.org/10.1007/s00404-023-07281-5

12. McGee, A.E.; Hawco, S.; Bhattacharya, S.; Hanley, S.J.B.; Cruickshank, M.E. Alternatives to surveillance for persistent human papillomavirus after a positive cervical screen: A systematic review and meta-analysis. Eur. J. Obstet. Gynecol. Reprod. Biol. 2024, 302, 332–338. https://doi.org/10.1016/j.ejogrb.2024.09.019

21. Numminen, E.; Leino, A.; Karhula, Q.; Fonagy, V.; Seiskari, T.; Paloniemi, M.; Kholová, I.; Kares, S.; Louvanto, K. Genotype and age shape the risk of persistent high-risk human papillomavirus infections. Am. J. Obstet. Gynecol. 2026, 235, 114–122. https://doi.org/10.1016/j.ajog.2026.02.038

22. Demarco, M.; Hyun, N.; Carter-Pokras, O.; Raine-Bennett, T.R.; Cheung, L.; Chen, X.; et al. A study of type-specific HPV natural history and implications for contemporary cervical cancer screening programs. eClinicalMedicine 2020, 22, 100293. https://doi.org/10.1016/j.eclinm.2020.100293

23. Rodríguez, A.C.; Schiffman, M.; Herrero, R.; Hildesheim, A.; Bratti, C.; Sherman, M.E.; et al. Longitudinal study of human papillomavirus persistence and cervical intraepithelial neoplasia grade 2/3: Critical role of duration of infection. J. Natl. Cancer Inst. 2010, 102, 315–324. https://doi.org/10.1093/jnci/djq001

25. Chen, H.-C.; Schiffman, M.; Lin, C.-Y.; Pan, M.-H.; You, S.-L.; Chuang, L.-C.; et al. Persistence of type-specific human papillomavirus infection and increased long-term risk of cervical cancer. J. Natl. Cancer Inst. 2011, 103, 1387–1396. https://doi.org/10.1093/jnci/djr283

27. Castle, P.E.; Rodríguez, A.C.; Burk, R.D.; Herrero, R.; Wacholder, S.; Alfaro, M.; et al. Short term persistence of human papillomavirus and risk of cervical precancer and cancer: Population based cohort study. BMJ 2009, 339, b2569. https://doi.org/10.1136/bmj.b2569

28. Jia, H.; Ding, L.; Han, Y.; Lyu, Y.; Hao, M.; Tian, Z.; et al. Genotype-specific distribution and change of high-risk human papillomavirus infection and the association with cervical progression risk in women with normal pathology and abnormal cytology in a population-based cohort study in China. J. Cancer 2021, 12, 4379–4388. https://doi.org/10.7150/jca.57993

29. Del Mistro, A.; Mancuso, P.; Carozzi, F.; De Marco, L.; Bisanzi, S.; Pompeo, G.; et al. Impact of differentiating between persistent and new infections on colposcopy referral in HPV-positive triage-negative women: Results from the NTCC2 study. Infect. Agent. Cancer 2025, 20, 84. https://doi.org/10.1186/s13027-025-00713-8

31. Polman, N.J.; Veldhuijzen, N.J.; Heideman, D.A.M.; Snijders, P.J.F.; Meijer, C.J.L.M.; Berkhof, J. HPV-positive women with normal cytology remain at increased risk of CIN3 after a negative repeat HPV test. Br. J. Cancer 2017, 117, 1557–1561. https://doi.org/10.1038/bjc.2017.309

33. Wentzensen, N.; Walker, J.L.; Gold, M.A.; Smith, K.M.; Zuna, R.E.; Mathews, C.; et al. Multiple biopsies and detection of cervical cancer precursors at colposcopy. J. Clin. Oncol. 2015, 33, 83–89. https://doi.org/10.1200/JCO.2014.55.9948

34. Dai, W.; Wang, T.; Chen, L.; Qiu, Z.; Chen, P.; Chen, D. Immediate risk of cervical intraepithelial neoplasia and diagnostic value of colposcopy among cytology-negative women with oncogenic HPV: A retrospective study. BMC Women's Health 2024, 24, 419. https://doi.org/10.1186/s12905-024-03258-x

35. Li, X.; Zhao, Y.; Xiang, F.; Zhang, X.; Chen, Z.; Zhang, M.; Kang, X.; Wu, R. Evaluation of the diagnostic performance of colposcopy in the detection of cervical high-grade squamous intraepithelial lesions among women with transformation zone type 3. BMC Cancer 2024, 24, 381. https://doi.org/10.1186/s12885-024-12156-2

38. Liu, A.H.; Walker, J.; Gage, J.C.; Gold, M.A.; Zuna, R.; Dunn, S.T.; et al. Diagnosis of cervical precancers by endocervical curettage at colposcopy of women with abnormal cervical cytology. Obstet. Gynecol. 2017, 130, 1218–1225. https://doi.org/10.1097/AOG.0000000000002330

40. Rusu, E.L.; Buciu, V.B.; Balan, L.; Serban, D.M.; Chiriac, J.; Chiriac, V.D. Additional diagnostic yield of endocervical curettage in type 3 transformation zone for high-grade cervical lesions: A retrospective analysis by human papillomavirus genotype. Diagnostics 2026, 16, 1220. https://doi.org/10.3390/diagnostics16081220

52. Manley, K.; Patel, A.; Pawade, J.; Glew, S.; Hunt, K.; Villeneuve, N.; et al. The use of biomarkers and HPV genotyping to improve diagnostic accuracy in women with a transformation zone type 3. Br. J. Cancer 2022, 126, 91–99. https://doi.org/10.1038/s41416-021-01539-y

70. Gilham, C.; Sargent, A.; Crosbie, E.J.; Peto, J. Long-term risks of invasive cervical cancer following HPV infection: Follow-up of two screening cohorts in Manchester. Br. J. Cancer 2023, 128, 1933–1940. https://doi.org/10.1038/s41416-023-02227-9

71. Hildesheim, A.; Herrero, R.; Wacholder, S.; Rodriguez, A.C.; Solomon, D.; Bratti, M.C.; et al. Effect of human papillomavirus 16/18 L1 viruslike particle vaccine among young women with preexisting infection: A randomized trial. JAMA 2007, 298, 743–753. https://doi.org/10.1001/jama.298.7.743

72. Hildesheim, A.; Gonzalez, P.; Kreimer, A.R.; Wacholder, S.; Schussler, J.; Rodriguez, A.C.; et al. Impact of human papillomavirus (HPV) 16 and 18 vaccination on prevalent infections and rates of cervical lesions after excisional treatment. Am. J. Obstet. Gynecol. 2016, 215, 212.e1–212.e15. https://doi.org/10.1016/j.ajog.2016.02.021

73. Maiorano, M.F.P.; Cazzolla, A.; Maiorano, B.A.; Loizzi, V.; Cormio, G.; Colonna, G.; et al. Impact of HPV vaccine on CIN2+ recurrence after conization: A systematic review and meta-analysis of vaccination timing, valency and surgical margins. Front. Oncol. 2026, 16, 1748972. https://doi.org/10.3389/fonc.2026.1748972

74. Trimble, C.L.; Morrow, M.P.; Kraynyak, K.A.; Shen, X.; Dallas, M.; Yan, J.; et al. Safety, efficacy, and immunogenicity of VGX-3100, a therapeutic synthetic DNA vaccine targeting human papillomavirus 16 and 18 E6 and E7 proteins for cervical intraepithelial neoplasia 2/3: A randomised, double-blind, placebo-controlled phase 2b trial. Lancet 2015, 386, 2078–2088. https://doi.org/10.1016/S0140-6736(15)00239-1

75. Fonseca, B.O.; Possati-Resende, J.C.; Salcedo, M.P.; Schmeler, K.M.; Accorsi, G.S.; Fregnani, J.H.T.G.; et al. Topical imiquimod for the treatment of high-grade squamous intraepithelial lesions of the cervix: A randomized controlled trial. Obstet. Gynecol. 2021, 137, 1043–1053. https://doi.org/10.1097/AOG.0000000000004384

76. Rahangdale, L.; Lippmann, Q.K.; Garcia, K.; Budwit, D.; Smith, J.S.; Van Le, L. Topical 5-fluorouracil for treatment of cervical intraepithelial neoplasia 2: A randomized controlled trial. Am. J. Obstet. Gynecol. 2014, 210, 314.e1–314.e8. https://doi.org/10.1016/j.ajog.2013.12.042

77. Van Damme, P.; Bouillette-Marussig, M.; Hens, A.; De Coster, I.; Depuydt, C.; Goubier, A.; et al. GTL001, a therapeutic vaccine for women infected with human papillomavirus 16 or 18 and normal cervical cytology: Results of a phase I clinical trial. Clin. Cancer Res. 2016, 22, 3238–3248. https://doi.org/10.1158/1078-0432.CCR-16-0085

78. Garcia, F.A.R.; Cornelison, T.; Nuño, T.; Greenspan, D.L.; Byron, J.W.; Hsu, C.-H.; et al. Results of a phase II randomized, double-blind, placebo-controlled trial of Polyphenon E in women with persistent high-risk HPV infection and low-grade cervical intraepithelial neoplasia. Gynecol. Oncol. 2014, 132, 377–382. https://doi.org/10.1016/j.ygyno.2013.12.034

79. Ou, Y.-C.; Fu, H.-C.; Tseng, C.-W.; Wu, C.-H.; Tsai, C.-C.; Lin, H. The influence of probiotics on genital high-risk human papilloma virus clearance and quality of cervical smear: A randomized placebo-controlled trial. BMC Women's Health 2019, 19, 103. https://doi.org/10.1186/s12905-019-0798-y

80. Laurie, C.; El-Zein, M.; Botting-Provost, S.; Tota, J.E.; Tellier, P.-P.; Coutlée, F.; et al. Efficacy and safety of a self-applied carrageenan-based gel to prevent human papillomavirus infection in sexually active young women (CATCH study): An exploratory phase IIB randomised, placebo-controlled trial. eClinicalMedicine 2023, 60, 102038. https://doi.org/10.1016/j.eclinm.2023.102038

81. Laurie, C.; El-Zein, M.; Tota, J.E.; Tellier, P.-P.; Coutlée, F.; Burchell, A.N.; et al. Impact of a carrageenan gel on viral load of genital human papillomavirus infections in sexually active women: Findings from the Carrageenan-gel Against Transmission of Cervical Human papillomavirus (CATCH) trial. J. Med. Virol. 2024, 96, e29604. https://doi.org/10.1002/jmv.29604

82. Serrano, L.; López, A.C.; González, S.P.; Palacios, S.; Dexeus, D.; Centeno-Mediavilla, C.; et al. Efficacy of a *Coriolus versicolor*-based vaginal gel in women with human papillomavirus-dependent cervical lesions: The PALOMA study. J. Low. Genit. Tract Dis. 2021, 25, 130–136. https://doi.org/10.1097/LGT.0000000000000596

83. Sánchez Díaz, E.; Levin, G.; Fernandes, A.; Fletcher, A.; Pareja, R. Clinical evidence on a *Coriolus versicolor*-based vaginal gel for HPV-related cervical disease: A narrative review. Chin. Clin. Oncol. 2026, 15, 9. https://doi.org/10.21037/cco-25-100

84. Yan, L.; Yin, H.; Shi, L.; Ding, B.; Liu, C.; Wang, Y.; Fu, C. Efficacy and safety of PHPV fusion factor vaginal gel (PHPV®) in clearing cervical persistent high-risk human papillomavirus infection: A randomized controlled clinical trial. Drug Des. Devel. Ther. 2026, 20, 542859. https://doi.org/10.2147/DDDT.S542859

85. Hecken, J.M.; Tempfer, C.B.; Klaus, K.; Scheich, J.; Rezniczek, G.A. Silicon dioxide vaginal gel and short-term high-risk human papillomavirus DNA detectability: A randomized controlled trial. Acta Obstet. Gynecol. Scand. 2026, advance online publication. https://doi.org/10.1111/aogs.70332

86. Chen, M.; Wang, X.; Abudurehemu, S.; Li, L.; Chen, C. Thermal ablation plus HPV-specific IgY vaginal gel versus watchful waiting in women with histologically confirmed CIN1 and persistent HPV: A prospective observational cohort study. BMC Women's Health 2026, 26, 299. https://doi.org/10.1186/s12905-026-04366-6

87. Denecke, A.; Emar, D.; Hillemanns, P.; Ramachandran, D.; Brüning, B.; Jentschke, M. Diagnostic cervical excision in patients with HPV positivity, cytological abnormalities and preoperative cervical stenosis. BMC Women's Health 2024, 24, 411. https://doi.org/10.1186/s12905-024-03195-9

95. Özen, S.; Güner Özen, E.; Özuyar Şimşek, G.; Arzik, A.O.; Karaoğlu, S.; Akdemir, C.; et al. HPV persistence and CIN2+ detection in women with normal cytology and non-16/18 high-risk HPV positivity. BMC Women's Health 2026, 26, 98. https://doi.org/10.1186/s12905-026-04278-5

100. Caeiro, V.; Nunes, S.; Esteves, B.; Moutinho-Fonseca, J. Repeated positive cervical HPV testing and absent or minor cytology abnormality at Pap smear: What is the next step? Asian Pac. J. Cancer Prev. 2021, 22, 1907–1912. https://doi.org/10.31557/APJCP.2021.22.6.1907
